# Engineering Oncolytic Virus‐Armed Macrophages for Enhanced Cancer Immunotherapy

**DOI:** 10.1002/advs.75406

**Published:** 2026-04-20

**Authors:** Jilong Wang, Ning Lu, Zhuo Yan, Luyi Ye, Luyao Bai, Shanshan Yuan, Yuting Zhu, Yiru Xiong, Yongheng Bai, Yaping Chen, Junjie Deng

**Affiliations:** ^1^ Joint Centre of Translational Medicine The First Affiliated Hospital of Wenzhou Medical University Wenzhou Medical University Wenzhou Zhejiang China; ^2^ Joint Centre of Translational Medicine Wenzhou Institute University of Chinese Academy of Sciences Wenzhou Zhejiang China; ^3^ Oujiang Laboratory (Zhejiang Lab for Regenerative Medicine Vision and Brain, Health) Wenzhou Zhejiang China; ^4^ Zhejiang Key Laboratory of Intelligent Cancer Biomarker Discovery and Translation The First Affiliated Hospital Wenzhou Medical University Wenzhou Zhejiang China; ^5^ Monash Institute of Pharmaceutical Sciences Monash University Parkville Victoria Australia

**Keywords:** anti‐tumor, armored macrophage, immunotherapy, Oncolytic adenovirus

## Abstract

Macrophage‐based immunotherapy holds great promise for solid tumors but is limited by impaired phagocytosis and unstable M1‐type polarization due to immunosuppressive tumor microenvironments. Here, we develop engineered macrophages by conjugating oncolytic adenovirus (OA)‐loaded zeolitic imidazolate framework‐8 (ZIF‐8) onto macrophage surfaces via bioorthogonal chemistry (ZIFOA‐M). This platform leverages localized viral delivery to selectively downregulate “don't eat me” signals (CD47 and CD24) on tumor cells, thereby restoring macrophage phagocytic capacity. Moreover, viral replication within tumors induces immunogenic cell death, releasing major damage‐associated molecular patterns (DAMPs) that sustain M1‐type polarization and promote durable antitumor immunity. ZIFOA‐M also enhances tumor antigen presentation, eliciting robust tumor‐specific T cell responses. Our strategy integrates phagocytosis restoration, microenvironment remodeling, and adaptive immune activation in a single, localized platform. ZIFOA‐M offers a complementary and translatable approach to overcome key barriers in macrophage‐based cancer immunotherapy.

## Graphical abstract

ZIFOA‐M is engineered by conjugating oncolytic adenovirus‐loaded ZIF‐8 nanoparticles onto macrophage surfaces via bioorthogonal chemistry. Upon tumor infiltration, the platform releases OA to downregulate CD47/CD24 on tumor cells, restoring macrophage phagocytosis. Concurrently, OA induces immunogenic cell death, releasing DAMPs to sustain M1 polarization. These events enhance antigen presentation and prime robust CD8^+^ T cell responses.

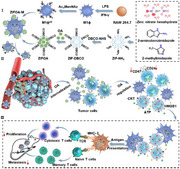

## 1 Introduction

Macrophage‐based immunotherapy has emerged as a promising strategy for treating solid tumors by harnessing the innate tumor‐homing capability of macrophages and hitchhiking immunomodulatory compounds [1, 2, 3, 4]. Although numerous studies have indicated the excellent tumor delivery capabilities of macrophages in enhancing the efficacy of tumor immunotherapy [5, 6, 7], the innate phagocytic capacity of macrophages and their subsequent ability to activate the adaptive immune system have not been fully exploited [8, 9]. These limitations primarily arise from the complex immunosuppressive mechanisms orchestrated by both tumor cells and the tumor microenvironment (TME). Tumor cells frequently overexpress inhibitory “don't eat me” signals, such as cluster of differentiation 47 (CD47), on their surface, which binds to the SIRPα receptor on macrophages, effectively inhibiting phagocytosis [10, 11]. Additionally, various soluble factors present in the TME‐including cytokines like interleukin‐4 (IL‐4), IL‐10, IL‐13, and transforming growth factor‐β (TGF‐β)‐promote the polarization of macrophages toward an anti‐inflammatory M2‐like phenotype, which is associated with tumor progression and immune suppression [12, 13, 14]. Collectively, these mechanisms impair the ability of macrophages to engulf tumor cells and efficiently present tumor antigens to T cells, thereby hindering the initiation of a robust adaptive immune response. Addressing these challenges necessitates innovative approaches to enhance macrophage persistence, polarization stability, and synergistic immunomodulation, ultimately unlocking the full potential of macrophage‐based immunotherapy.

Recent studies have sought to address these limitations through various strategies; however, critical gaps remain. For example, to counteract “don't eat me” signals, researchers have combined macrophages with CD47 blockade [10, 15, 16, 17]. Bian et al. demonstrated that anti‐CD47 antibodies could synergize with macrophages to enhance phagocytosis in solid tumors [18]. Nonetheless, systemic inhibition of CD47 poses risks of hematologic toxicity, which limits its clinical applicability [19, 20, 21]. Alternatively, engineering immune cells to secrete SIRPα decoys has shown promise in preclinical models [22], although this approach does not fully mitigate the immunosuppressive TME that still dampens macrophage function. Maintaining a pro‐inflammatory M1 polarization within the TME is crucial for sustained antitumor activity [14, 23, 24, 25]. Some studies have stimulated immunostimulatory domains [e.g., toll‐like receptor‐4 (TLR4) or myeloid differentiation primary response protein 88 (MyD88)] of macrophages to promote pro‐inflammatory signaling, while others have co‐administered interferon‐γ (IFN‐γ) to reinforce M1 phenotypes [26, 27, 28]. Although these strategies can delay M2 conversion, they often necessitate repeated dosing and fail to achieve durable remodeling of the TME. Moreover, excessive inflammation resulting from constitutive M1 signaling may exacerbate toxicity [29, 30]. Current strategies tend to address individual limitations but lack a cohesive approach to simultaneously enhance phagocytosis, sustain M1 polarization, and amplify adaptive immunity.

Recently, oncolytic virus (OV) therapy has demonstrated immune‐amplifying effects in cancer treatment and can effectively enhance tumor immunotherapy as part of a combinatorial approach [31, 32]. Evidence that OVs alter tumor cell transcription, metabolism, and immunogenicity [33, 34, 35] during replication implies the potential of OV‐based bioactive materials to downregulate “don't eat me” signals, such as CD47 and CD24, on these tumor cells. This modulation significantly enhanced the phagocytic capacity of macrophages. In contrast to antibody blockade, OV‐mediated modulation is localized, thereby reducing the risks of systemic toxicity. Furthermore, OVs have been shown to induce immunogenic cell death (ICD), resulting in the release of damage‐associated molecular patterns (DAMPs) and pathogen‐associated molecular patterns (PAMPs), which activate TLR/STING pathways in macrophages and reinforce M1 polarization [36, 37]. Unlike cytokine priming, this autocrine stimulation is self‐sustaining within tumors. Additionally, OV‐mediated lysis releases tumor‐associated antigens, which are efficiently captured and presented by macrophages. OVs thus differ fundamentally from non‐biological immunomodulators or particle‐based systems. As bioactive agents, their combination with macrophages depends not only on tumor‐targeting delivery but also on synergistic modulation of intrinsic macrophage functions, such as tumor phagocytosis and immune activation. Consequently, OV‐macrophage synergy represents a promising strategy to enhance sustained phagocytic activity against tumors and promote robust adaptive antitumor immunity. Drug delivery carriers have demonstrated increasing advantages in antitumor therapy [38, 39]. Among them, metal‐organic frameworks are an emerging class of inorganic crystalline porous materials, constructed from metal nodes and organic ligands via coordination bonds. Zeolitic imidazolate framework‐8 (ZIF‐8) has also shown promising efficacy as a delivery vehicle for active agents such as viruses [40]. ZIF‐8 features highly tunable porosity and acid‐labile coordination bonds, enabling high‐capacity loading of diverse bioactive cargos and achieving rapid release in response to the acidic tumor microenvironment.

**SCHEME 1:**
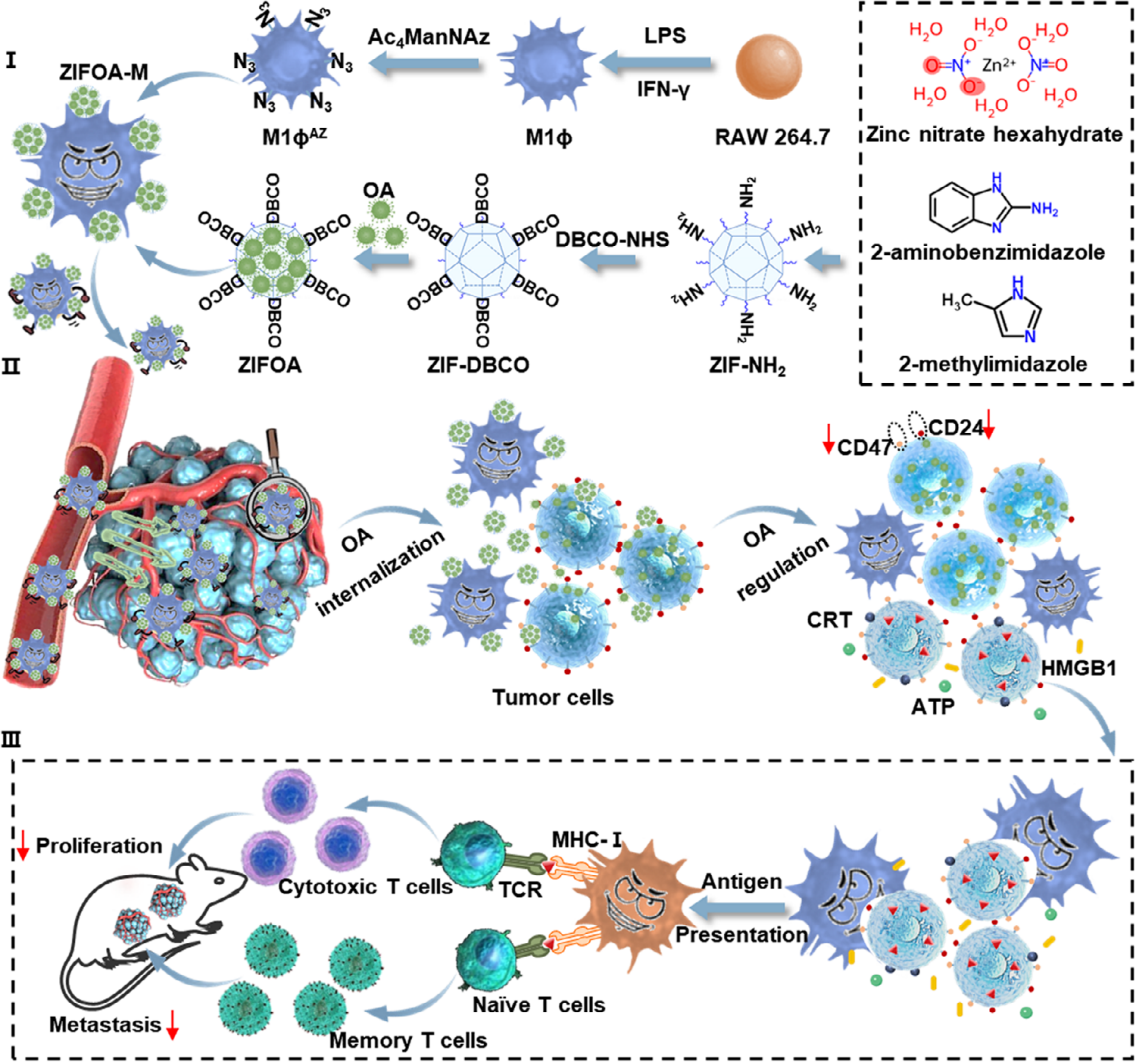
Schematic illustration of the ZIFOA‐M system and its mechanisms for enhanced cancer immunotherapy. (I) Preparation of ZIFOA‐M via bioorthogonal conjugation of OA‐loaded ZIF‐8 to macrophage surfaces. (II) OA‐mediated downregulation of “don't eat me” signals (CD47/CD24) on tumor cells and induction of immunogenic cell death (ICD). (III) Enhanced tumor cell phagocytosis by macrophages; Promotion of antigen presentation and robust activation of tumor‐specific CD8^+^ T cell responses.

In this study, we developed an OA‐armed macrophage by conjugating oncolytic adenovirus (OA)‐loaded ZIF‐8 to the surfaces of macrophages (designated as ZIFOA‐M) using bioorthogonal chemistry. Our results demonstrate that the ZIFOA‐M system effectively downregulates “don't eat me” signals, such as (CD47 and CD24), significantly enhancing the phagocytic capacity of macrophages against tumor cells. Furthermore, this approach has the potential to reshape the immunosuppressive TME through virus‐induced ICD, which releases DAMPs and PAMPs that help maintain stable M1 polarization in the armored macrophages. Additionally, ZIFOA‐M acts as a potent antigen source, promoting efficient antigen presentation and leading to robust activation of tumor‐specific T cell responses (Scheme 1). This strategy effectively addresses the limitations of current macrophage‐based immunotherapy by simultaneously overcoming phagocytosis deficits and polarization instability. ZIFOA‐M, therefore, serves as a complementary alternative to macrophage‐based approaches, enhancing their therapeutic potential in clinical.

## 2 Results and Discussions

### 2.1 Preparation and Characterization of ZIFOA‐M

**FIGURE 1:**
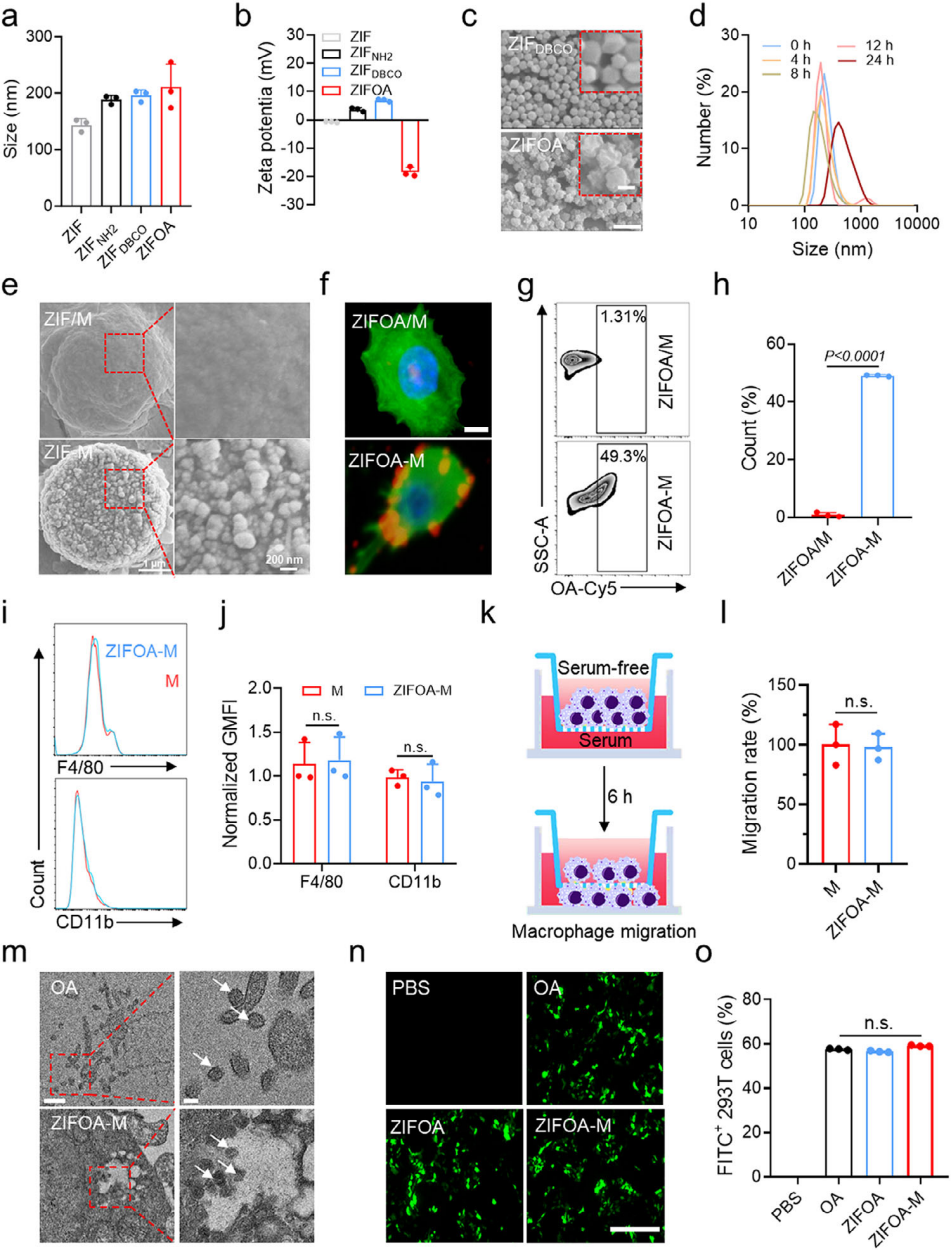
Characterization of ZIFOA‐M. Size (a) and zeta potential (b) distribution of ZIF, ZIF_NH2_, ZIF_DBCO_ and ZIFOA. (c) SEM images of ZIF_DBCO_ and ZIFOA morphology. Long scale bar: 500 nm; Short scale bar: 200 nm. (d) Size distribution of ZIFOA under acid conditions (pH 6.5). SEM (e) and CLSM (f) observations of ZIFOA/M and ZIFOA‐M. CLSM scale bar: 5 µm. Flow analysis of the binding efficiency of ZIFOA to macrophages (g, h) and the expression levels of surface markers F4/80 and CD11b (i, j) on macrophages. (k, l) Schematic of macrophage migration in a Transwell system. (m) TEM images of free OA and OA isolated from ZIFOA‐M morphology. (n, o) CLSM images and quantification analyses of different OA formulations‐infected 293T cells. Scale bar: 50 µm. Data were expressed as mean ± standard deviations (SD) (*n* = 3).

To fabricate OV‐armed macrophage, bioorthogonal approach was employed to conjugate ZIF particles loaded with OA onto the surface of macrophages. ZIF particles with differing levels of amino functional groups (ZIF_NH2_) were synthesized, with their sizes consistently regulated to approximately 100 nm (Figure S1). SEM analysis confirmed that all particles exhibited spherical morphology (Figure S2). FTIR spectroscopy verified the successful functionalization of amino groups on the surface of the ZIF particles. Among the different ratios tested, the particles prepared with a molar ratio of diaminobenzimidazole to dimethylimidazole of 2: 3 exhibited the highest content of amino groups (Figure S3). Consequently, this molar ratio was employed for the synthesis of ZIF particles in subsequent experiments. ZIF_NH2_ was then reacted with DBCO‐NHS, thereby functionalizing its surface with DBCO. DLS analysis revealed that modification of the ZIF surface with DBCO resulted in an increase in particle size of approximately 7.6 ± 4.3 nm. Subsequent loading of OA further increased the particle size by about 66.3 ± 8.7 nm (Figure 1a). Additionally, the surface potential of the ZIF particles shifted from 6.92 ± 0.34 mV to −18.43 ± 1.27 mV following these modifications (Figure 1b). SEM imaging further revealed that ZIF particles loaded with OA exhibited significantly greater surface roughness than their unloaded counterparts (Figure 1c). Together, these results confirmed the successful loading of OA onto ZIF.

To evaluate the acid‐responsive properties of the modified ZIF particles, they were incubated in an acidic buffer (pH 6.5). DLS measurements revealed a significant change in particle size over time (Figure 1d), indicating the gradual degradation of ZIFOA in acidic buffer, accompanied by substantial OA release (Figure S4). To assess the conjugation of ZIF particles to the surface of macrophages, SEM imaging demonstrated that the presence of numerous particles adhered to the surface of ZIF‐M, thereby providing evidence of successful conjugation. In contrast, the mixture of ZIF and macrophages (ZIF/M) did not retain many particles on the surface of cells, and overall, it appeared slightly smooth (Figure 1e). To quantify the efficiency of conjugation, OA was fluorescently labeled with Cy5 (OA‐Cy5). CLSM measurements detected the red fluorescence signals from OA‐Cy5 distributed across the macrophage surface. However, the mixture of ZIFOA and macrophages (ZIFOA/M) showed no intense red fluorescence signal on the macrophage surfaces (Figure 1f). Flow cytometry analysis quantitatively confirmed a high OA‐Cy5 positive rate of 49.3% (Figure 1g,h). To confirm that the preparation process did not compromise the intrinsic properties of OA or M1 macrophages, a series of functional assays was conducted. Comparative analysis of the expression levels of characteristic macrophage surface markers (F4/80 and CD11b) between normal macrophages (M) and ZIFOA‐M revealed that the surface conjugation of ZIFOA did not affect the expression of these markers (Figure 1i,j). Furthermore, given the critical role of macrophage migratory capacity, a transwell migration assay was conducted to compare the migration abilities of normal macrophages and ZIFOA‐M. The results demonstrated no significant difference in the number of migrated cells between the two groups (Figure 1k,l). TEM images revealed that both the free OA and the OA separated from ZIFOA‐M exhibited spherical morphologies with no significant differences (Figure 1m). Additionally, when co‐incubated with 293T cells, the infectivity of the conjugated OA was comparable to that of free OA in the control group, with no statistically significant differences observed (Figure 1n,o). These results indicate that neither ZIF‐8 encapsulation nor subsequent conjugation to macrophages compromised the structural integrity or infectivity of the oncolytic virus. Therefore, these findings demonstrated that we successfully fabricated an armored macrophage system (ZIFOA‐M) based on OA‐loaded ZIF particles conjugated to the M1 macrophage surface using a bioorthogonal approach. Importantly, this preparation process did not impair the intrinsic functionalities of either the OA or the M1 macrophages.

### 2.2 Enhanced Accumulation of ZIFOA‐M in Tumor

**FIGURE 2:**
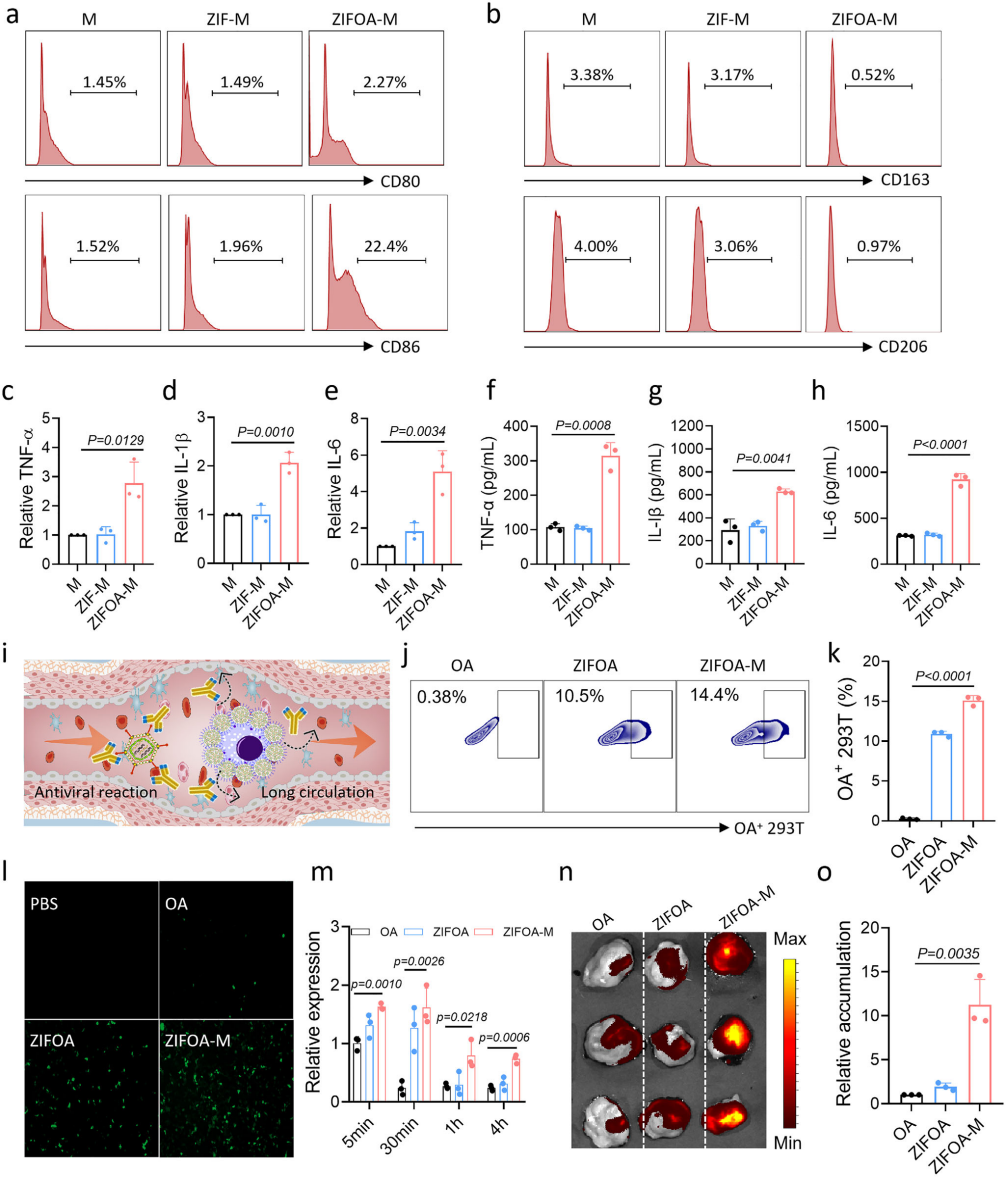
Enhanced tumor‐targeting function of ZIFOA‐M. (a, b) Flow cytometry analysis of M1 (CD80, CD86) and M2 (CD163, CD206) macrophage polarization markers in different formulations. qPCR (c–e) and ELISA (f–h) quantitative analysis of inflammatory cytokine secretion (TNF‐α, IL‐1β, and IL‐6) in macrophages. (i) Schematic of OA clearance by innate immunity and ZIFOA‐M's protective mechanism. (j–l) Neutralization antibody assays showing ZIFOA‐M maintains 293T cell infectivity after incubation with high‐titer neutralizing antiserum. Scale bar: 100 µm. (m) Pharmacokinetic profiles of different formulations in blood. (n, p) Accumulation of different formulations in tumors. Data were expressed as mean ± standard deviations (SD) (*n* = 3). [Correction added on 15 June 2026, after first online publication: Figure 2 is updated in this version.]

To investigate the biological function of ZIFOA‐M, we analyzed the maintenance of its anti‐tumor phenotype. Flow cytometry was employed to examine the phenotype of macrophages across different formulations. In ZIFOA‐M, the expression of CD80 and CD86 molecules, which were markers of the M1 phenotype, was markedly elevated compared to the M and ZIF‐M, with CD86 expression being approximately 15 times higher than that in the unconjugated macrophage (M) and ZIF‐conjugated macrophage (ZIF‐M) groups (Figure 2a). In contrast, the expression of CD163 and CD206 molecules, markers of the M2 phenotype, was reduced by approximately 85% and 76%, respectively, compared to the M group (Figure 2b). Quantitative flow cytometry data further indicated that loading OA promoted the polarization of macrophages towards the M1 phenotype. Furthermore, the macrophage supernatants were collected, and the expression levels of inflammatory factors (TNF‐α, IL‐1β, and IL‐6) were quantified via qPCR and ELISA. The results revealed that the expression of these inflammatory factors in macrophages within ZIFOA‐M group was significantly higher than in those from M and ZIF‐M groups (Figure 2c–h).

It is well‐established that OA typically induces innate antiviral immune responses in the bloodstream, leading to its rapid clearance [35] (Figure 2i). Our composite system of ZIFOA‐M was specifically designed to overcome this limitation. Neutralization antibody assays demonstrated that ZIFOA‐M effectively evaded antibody‐mediated neutralization in vitro (Figure 2j,k). When co‐incubated with high‐titer neutralizing antiserum, ZIFOA‐M maintained its infectivity towards 293T cells without significant reduction, whereas naked OA showed markedly diminished cellular infectivity (Figure 2l). This protective effect was further confirmed using a low‐concentration formulation, yielding consistent results (Figure S5). These findings collectively indicated that our composite system successfully preserved the inherent functionality of each component while effectively circumventing host clearance mechanisms. Therefore, ZIFOA‐M was expected to significantly prolong its circulation time in the bloodstream and enhance tumor accumulation. Pharmacokinetic studies demonstrated that while the OA concentration in blood declined rapidly in the naked OA group, ZIFOA‐M sustained significantly higher levels of OA in systemic circulation (Figure 2m). Additionally, L929 (normal mouse fibroblast) and 4T1 (mouse breast cancer) cells were co‐cultured in distinct regions of the same well with ZIFOA‐M formulations. Figure S6 demonstrated that preferential migration of ZIFOA‐M toward 4T1 cells, directly confirming its tumor‐targeting capability in vitro. Figure 2n,o showed that tumor accumulation of ZIFOA‐M in vivo was approximately 15‐fold higher compared to the naked OA. No significant differences in viral load were observed in other major organs (Figure S7), demonstrating its tumor‐targeting capability and safety. These results indicated that our engineered modification successfully preserved the inherent properties and biological functions of both M1 macrophages and OA, while effectively enhancing tumor‐targeting capability.

### 2.3 Penetration of ZIFOA‐M in Tumor

**FIGURE 3:**
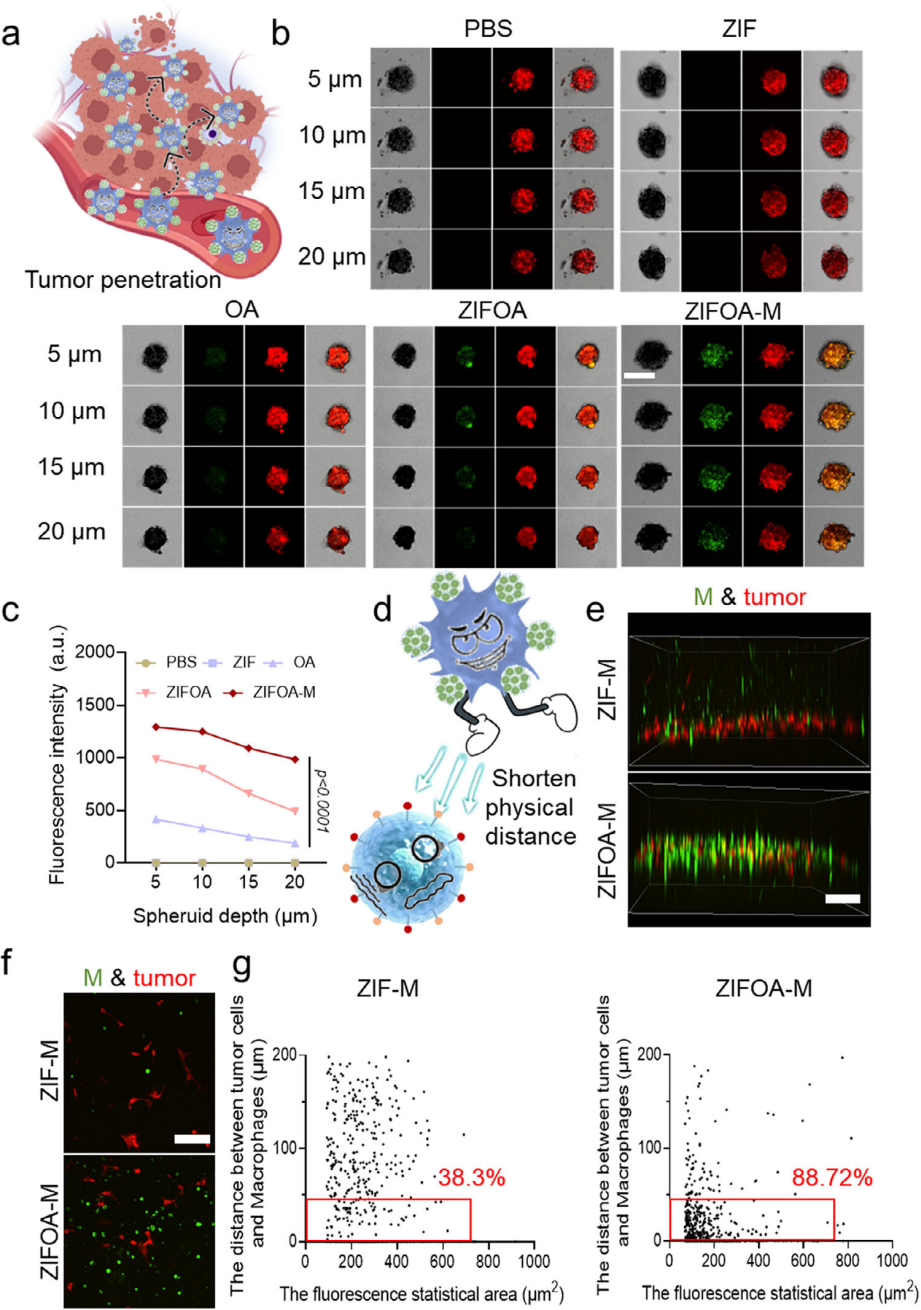
Penetration of ZIFOA‐M in tumor. (a) Schematic illustration of tumor spheroid penetration. Representative images (b) and quantitative analysis (c) showing the penetration profiles of different OA formulations in 3D tumor spheroids. Scale bar: 100 µm. (d) Spatial interactions between ZIFOA‐M and tumor cells. (e) 3D confocal reconstruction revealing enhanced tumor‐targeting capability of ZIFOA‐M compared to ZIF‐M controls. Scale bar: 500 µm. (f) High‐resolution confocal images showing dense peritumoral accumulation of ZIFOA‐M macrophages (green) surrounding tumor cells (red). Scale bar: 100 µm. (g) Tissue flow cytometry quantification of macrophage‐tumor cell proximity. Data were expressed as mean ± standard deviations (SD) (*n* = 3).

To investigate the intratumoral distribution of therapeutic agents (which must diffuse into/enter the tumor core to exert anti‐tumor effects), we evaluated the penetration capacity of various OA‐derived formulations (OA, ZIFOA, and ZIFOA‐M) within 3D tumor spheroids. We established a 3D multicellular tumor spheroid model with a diameter of 100 µm, composed of breast cancer cells expressing mCherry fluorescence. The OA used in this experiment was designed with GFP (GFP‐OA). GFP expression allowed for the fluorescence characterization of lateral spheroid sections using confocal laser microscopy, and tomographic images were acquired at fixed depth intervals of 5 µm (Figure 3a). As demonstrated in Figure 3b, ZIFOA‐M exhibited significantly enhanced tumor penetration efficiency compared to free OA and ZIFOA. Quantitative fluorescence analysis further confirmed the successful accumulation of OA within the tumor core (Figure 3c), suggesting ZIFOA‐M's superior ability to overcome diffusion barriers in solid tumors. To elucidate the interaction mode between ZIFOA‐M and tumor cells, we performed quantitative and qualitative analyses of the spatial proximity between this biomimetic system and tumor cells (Figure 3d). 3D confocal imaging revealed that ZIFOA‐M exhibited significantly enhanced tumor‐targeting capability compared to ZIF‐M (Figure 3e). Qualitative assessment of tumor cells‐macrophages confocal plane demonstrated that dense accumulation of macrophages surrounding tumor cells in the ZIFOA‐M group (Figure 3f). Tissue flow cytometry quantification further demonstrated that macrophages in the ZIFOA‐M group maintained substantially closer physical proximity to tumor cells, with 88.72% of macrophages localized within a 50 µm radius of tumor cells (Figure 3g). Notably, the number of macrophages within the critical 10 µm tumor cells interaction zone was threefold higher in ZIFOA‐M group compared to ZIF‐M group. Whole‐cell statistical analysis confirmed the superior tumor‐targeting efficiency of ZIFOA‐M (Figure S8). These results demonstrated that the intrinsic tumor‐targeting property of OA effectively guided macrophage trafficking to tumor cells, potentially enhancing their tumoricidal activity.

### 2.4 Tumor Suppression by ZIFOA‐M

**FIGURE 4:**
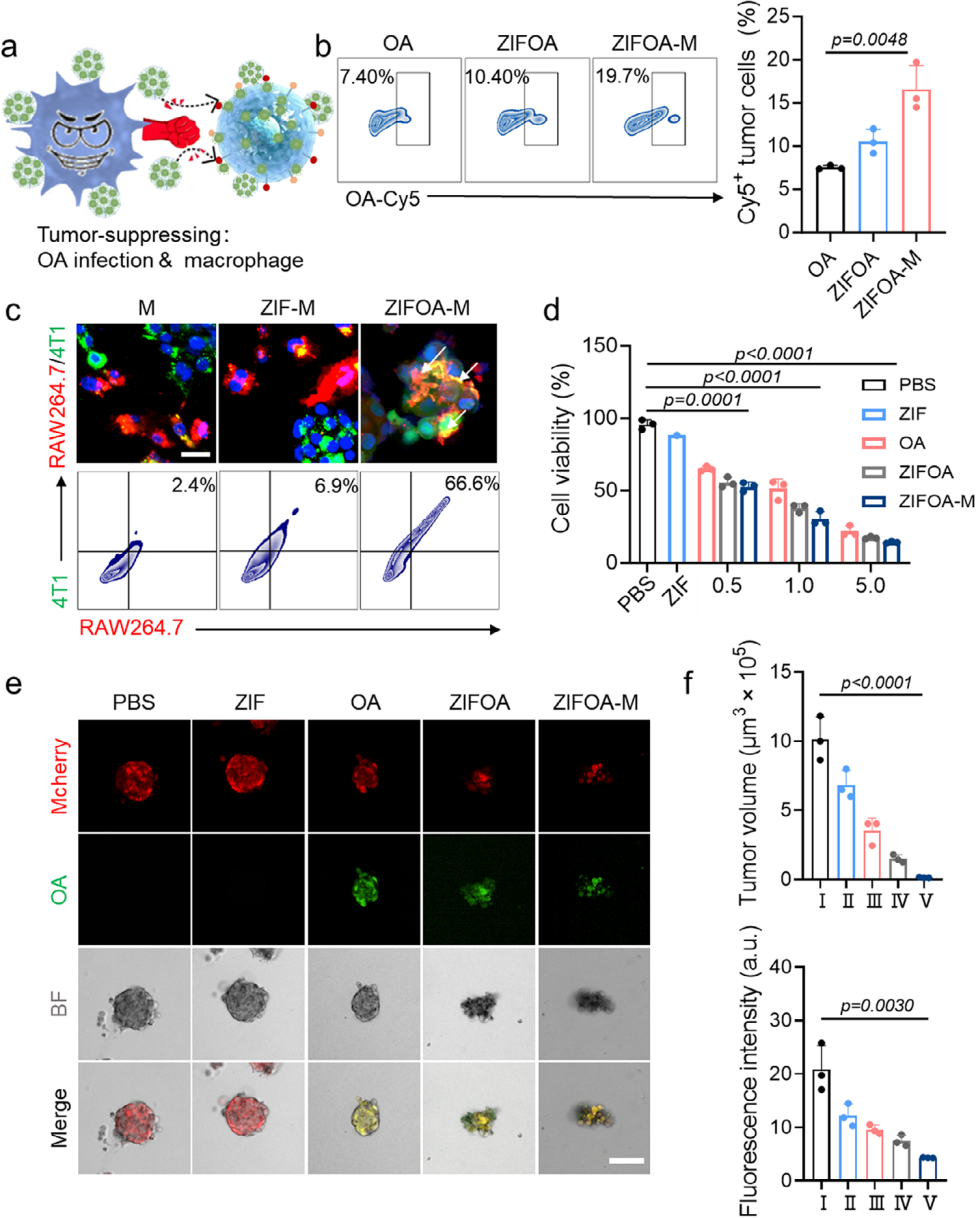
Antitumor efficacy of ZIFOA‐M. (a) Tumor inhibition through OA‐mediated tumor cell infection and macrophage activation. (b) Quantitative analysis of OA infection efficiency in tumor cells via flow cytometry. (c) CLSM images and flow cytometry analysis of phagocytosis of OA‐infected tumor cells by M1 macrophages. Scale bar: 25 µm. (d) Dose‐dependent tumor cell killing by different formulations. (e) 3D tumor spheroid assays of tumor growth inhibition. Scale bar: 100 µm. (f) Quantitative analysis of spheroid volume and fluorescence intensity. Data were expressed as mean ± standard deviations (SD) (*n* = 3).

To investigate whether ZIFOA‐M penetrating into tumor tissues could effectively suppress tumor cell growth, we examined the oncolytic activity of OA‐infected tumor cells and the tumoricidal effect by macrophages (Figure 4a). Following tail vein injection of different formulations into tumor‐bearing mice, tumor cells were isolated by flow cytometry sorting. Quantitative analysis of OA within tumor cells demonstrated that the OA infection rate in the ZIFOA‐M group was significantly increased to 19.7% compared to the OA control group (Figure 4b). Confocal microscopy observations revealed significant phagocytosis of tumor cells by oncolytic virus‐modified macrophages. The results showed that the phagocytic levels of normal macrophages and ZIF‐modified macrophages were only 2.4% and 6.9%, respectively. In contrast, the phagocytic capacity of oncolytic virus‐modified macrophages toward tumor cells reached as high as 66.6% (Figure 4c). These results indicated that OA infection facilitated subsequent macrophage‐mediated tumor cell clearance. Cell viability assays revealed that ZIFOA‐M exhibited potent tumor cell killing efficacy in a dose‐ and time‐dependent manner, while showing no cytotoxic effects on normal cells (Figure 4d and Figure S9). Three‐dimensional tumor spheroid assays using 4T1‐mCherry cells further demonstrated the superior antitumor activity of ZIFOA‐M, as evidenced by significant reduction in spheroid volume and quantitative fluorescence measurements compared to other treatment groups (Figure 4e,f). Collectively, these findings demonstrated that the ZIFOA‐M composite system effectively utilized OA to infect tumor cells, promoted M1 macrophage‐mediated phagocytosis and elimination of tumor cells.

### 2.5 The Mechanisms of Tumor Cell Death by ZIFOA‐M

**FIGURE 5:**
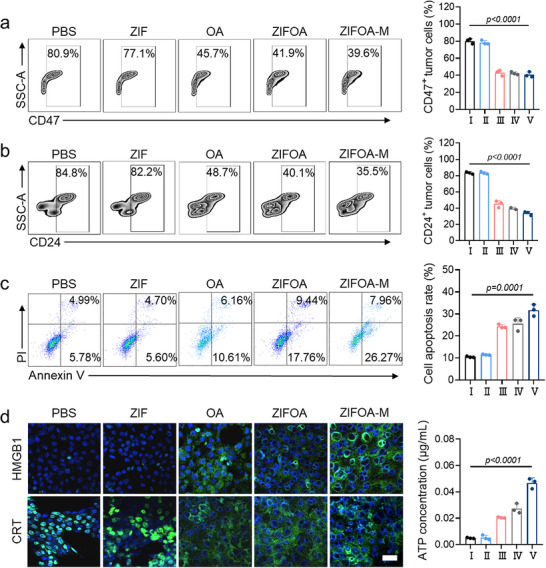
Mechanisms of tumor cell death by ZIFOA‐M. (a) Flow cytometry analysis showing CD47 expression levels on 4T1 cells after treatment with different formulations. (b) Flow cytometry analysis of CD24 expression on 4T1 cells post‐treatment. (c) Apoptosis assay demonstrating ZIFOA‐M induced tumor cell death. (d) CLSM images showing ICD markers: HMGB1 nuclear‐to‐cytoplasmic translocation, CRT membrane exposure, and extracellular ATP release. Scale bar: 25 µm. Data were expressed as mean ± standard deviations (SD) (*n* = 3).

The phagocytic process of macrophages is regulated by receptor‐ligand interactions, with the CD47‐SIRPα signaling pathway serving as a crucial phagocytic checkpoint [41, 42]. Tumor cells frequently overexpress CD47 to evade phagocytic clearance. Additionally, it was discovered that CD24, another “don't eat me” signal protein expressed by breast cancer cells, also mediates immune evasion from macrophage phagocytosis [43, 44]. Therefore, we sought to investigate whether ZIFOA‐M enhances macrophage phagocytic activity by downregulating CD47 and CD24 expression on tumor cells. When 4T1 cells were co‐cultured with different formulations, flow cytometry analysis revealed that ZIFOA‐M significantly reduced CD47 and CD24 expression on tumor cells. Notably, OA treatment alone decreased CD47 expression from 80.9% to 41.9% and CD24 expression from 84.4% to 35.5% on the tumor cell surface (Figure 5a,b). Furthermore, we performed quantitative real‐time PCR (qPCR) to examine the regulatory effect of ZIFOA‐M on the expression of CD24 and CD47 genes in tumor cells. The qPCR results showed that ZIFOA‐M also significantly downregulated the gene expression levels of both CD24 and CD47, a trend consistent with the findings obtained from flow cytometry analysis (Figure S10). These findings suggested that the enhanced phagocytic activity of macrophages against tumor cells was mediated by OA, which downregulated CD47 and CD24 expression, thereby attenuating immune evasion signals. Furthermore, our experiments demonstrated that the ZIFOA‐M composite system significantly promoted tumor cell apoptosis (Figure 5c). In‐depth analysis revealed that co‐culture with ZIFOA‐M induced ICD in tumor cells. Immunogenic cell death (ICD) is characterized by the release of damage‐associated molecular patterns (DAMPs), such as adenosine triphosphate (ATP) and high‐mobility group box 1 (HMGB1), from dying cells. The induction of ICD activates anti‐tumor immunity, thereby enhancing the efficacy of immunotherapy [45]. CLSM analysis confirmed the translocation of HMGB1 from the nucleus to the cytoplasm, CRT exposure from the endoplasmic reticulum to the cell membrane, and ATP release into the extracellular space (Figure 5d). Importantly, ZIFOA‐M induced more pronounced ICD hallmarks compared to other treatments. Collectively, these results indicated that ZIFOA‐M not only suppressed “don't eat me” signals (CD47 and CD24) on tumor cells but also triggered immunogenic cell death, ultimately facilitating M1 macrophage‐mediated phagocytosis of tumor cells.

### 2.6 Activation of T Cell‐Mediated Adaptive Immune Response

**FIGURE 6:**
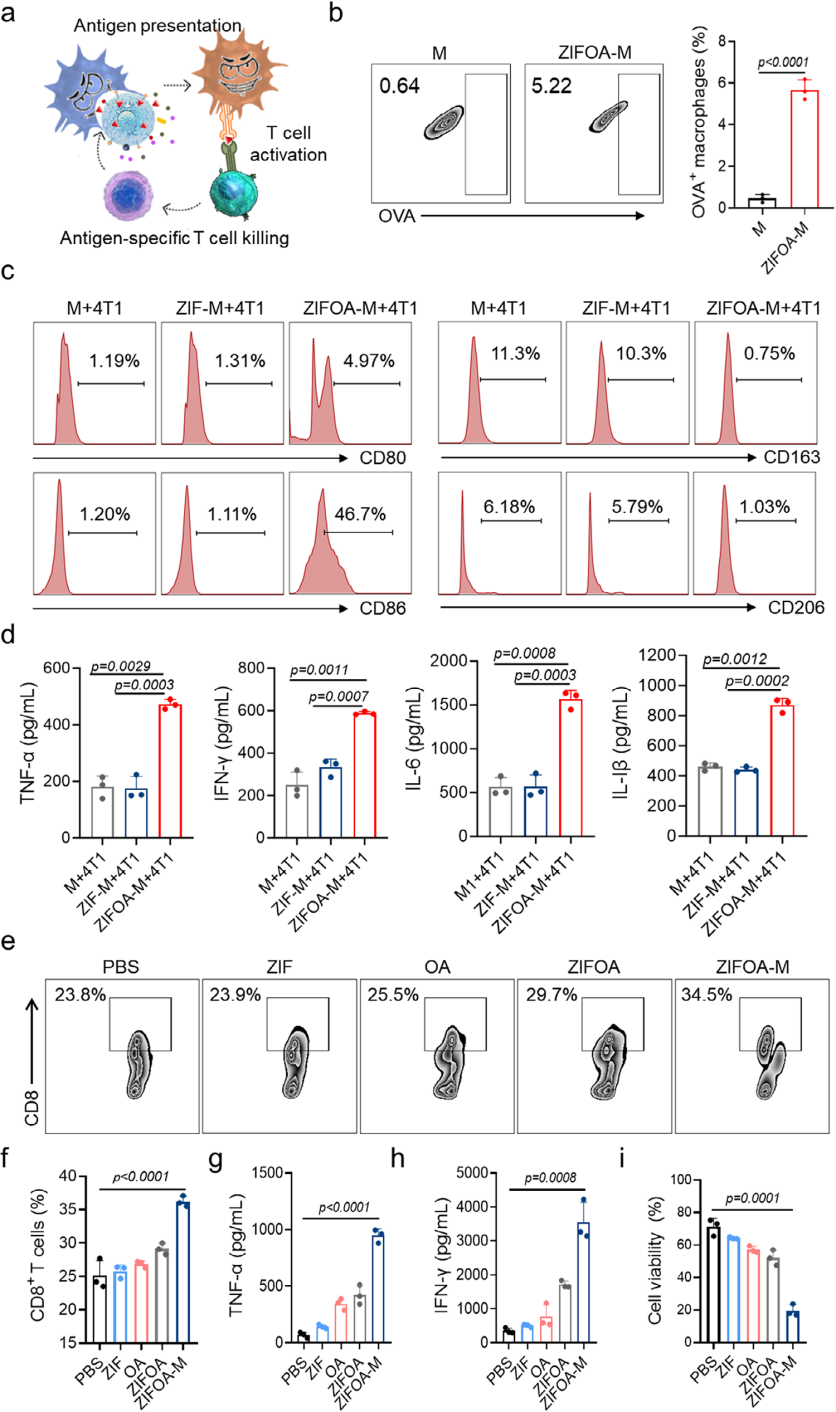
ZIFOA‐M enhanced antigen presentation and activated T cells‐mediated antitumor immunity. (a) Schematic of experimental design evaluating M1 macrophage‐mediated immune activation. (b) Flow cytometry analysis of OVA antigen presentation by ZIFOA‐M‐treated macrophages compared to unmodified M1 macrophages. (c) Representative flow plots and quantification of M1 surface markers (CD80^+^/CD86^+^) and M2 surface markers (CD163^+^/CD206^+^) after 4T1 co‐culture. (d) ELISA analysis of pro‐inflammatory cytokines (TNF‐α, IFN‐γ, IL‐6, and IL‐1β) in culture supernatants. (e,f) Flow cytometry analysis of increased CD8^+^ T cell populations in different formulation groups. (g,h) Cytokine production (TNF‐α and IFN‐γ) by activated T cells. (i) Tumor cell cytotoxicity of different formulations‐primed T cells. Data were expressed as mean ± standard deviations (SD) (*n* = 3).

To investigate the immunomodulatory effects following M1 macrophage‐mediated tumor cell phagocytosis, we systematically evaluated the induced T cell‐mediated adaptive immune responses. First, we examined the antigen‐presenting capacity of ZIFOA‐M (Figure 6a). We established a stable ovalbumin (OVA)‐expressing breast cancer cell line, 4T1‐OVA, to investigate the antigen presentation capacity of ZIFOA‐M. When 4T1‐OVA cells were co‐cultured with ZIFOA‐M, flow cytometry revealed an 8.2‐fold enhancement in OVA antigen presentation compared to unmodified M1 macrophages (Figure 6b), demonstrating that OA surface modification significantly potentiated M1 macrophage antigen‐presenting function. Although M1‐like macrophages possess antitumor properties, most tumor‐associated macrophages (TAMs) respond to signals from the TME and polarize toward a tumor‐promoting M2‐like phenotype, thereby facilitating rapid tumor progression [46, 47]. Maintenance of the pro‐inflammatory macrophage phenotype was crucial for sustained antitumor activity. Subsequent phenotypic characterization of macrophages after 4T1 co‐culture showed that ZIFOA‐M‐treated macrophages maintained robust M1 polarization, as evidenced by significant upregulation of M1 surface markers (CD80^+^/CD86^+^) and downregulation of M2 surface markers (CD163^+^/CD206^+^) (Figure 6c). Meanwhile, Figure 6d showed that the elevated secretion of pro‐inflammatory cytokines (TNF‐α, IFN‐γ, IL‐6, and IL‐1β) in culture supernatants. These findings indicated that ZIFOA‐M promoted an enhanced antitumor M1 phenotype within the tumor microenvironment. We further investigated T lymphocyte modulation by ZIFOA‐M‐activated macrophages. CD8^+^ T cell populations increased to 37.2% (1.5‐fold vs. PBS control) (Figure 6e,f). Treg populations decreased to 2.82% (0.2‐fold vs. PBS control) (Figure S11). Enhanced TNF‐α and IFN‐γ production by activated T cells (Figure 6g,h). Notably, in vitro tumor killing assays demonstrated that T cells primed by ZIFOA‐M‐treated macrophages exhibited superior cytotoxic activity against tumor targets, achieving maximal tumor cell elimination after 24 h co‐culture (Figure 6i). Collectively, these results demonstrated that ZIFOA‐M could sustain M1‐polarized macrophage states, enhanced tumor antigen presentation and promote antitumor CD8^+^ T cells responses.

### 2.7 Anti‐Tumor in Vivo

**FIGURE 7:**
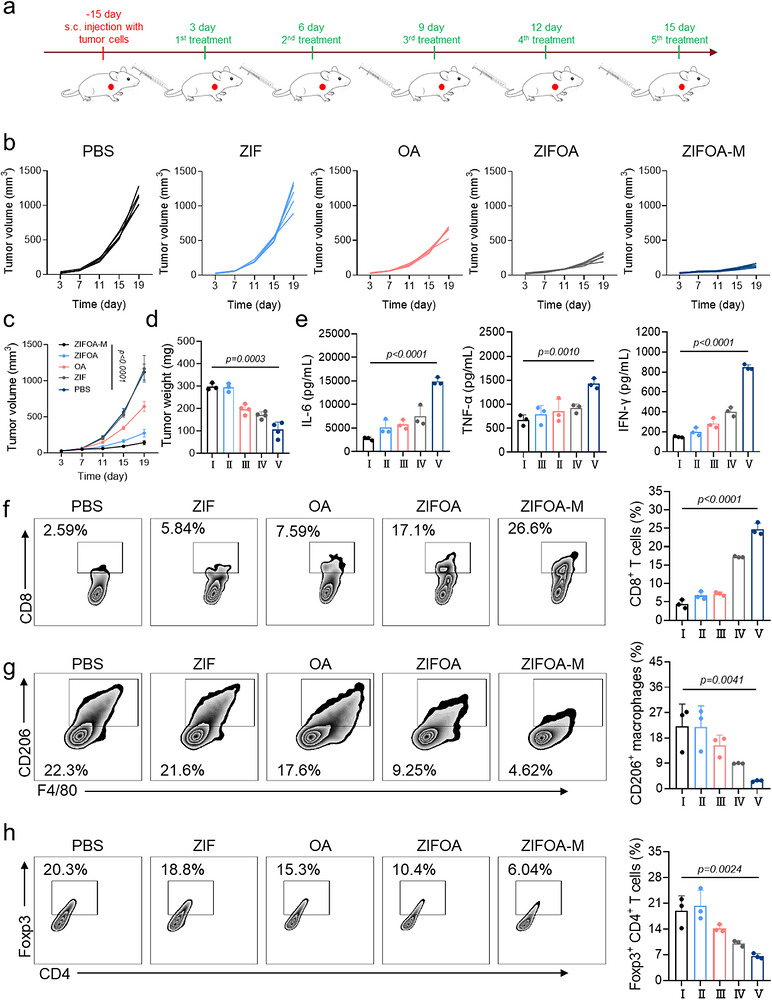
Anti‐tumor efficacy and immunomodulatory activity in an orthotopic 4T1 breast cancer model. (a) Schematic illustration of the experimental timeline and treatment regimen. (b,c) Tumor growth curves in different treatment groups. (d) Tumor weights of different formulations of treatments. (e) Evaluation of pro‐inflammatory cytokines (IL‐6, TNF‐α, and IFN‐γ) in treated tumors. (f–h) Flow cytometry analysis of CD8^+^ T cell infiltration, M2 macrophages, and Tregs populations in tumors. Data were expressed as mean ± standard deviations (SD) (*n* = 5).

To evaluate the antitumor efficacy of ZIFOA‐M, we established an orthotopic breast cancer model in mice by inoculating 4T1 cells into the mammary fat pad. As illustrated in Figure 7a, treatment with ZIFOA‐M demonstrated superior tumor growth suppression compared to control groups (PBS and ZIF alone) and the free OA group, which exhibited only marginal therapeutic effects (Figure 7b,c). Statistical results demonstrated that ZIFOA‐M achieved a high tumor suppression rate of 87.2% (Figure S12). Post‐treatment tumor weight measurements further confirmed the enhanced antitumor activity of ZIFOA‐M (Figure 7d). Throughout the treatment period, no significant changes in body weight were observed in the mice, indicating the relative safety of our therapeutic system (Figure S13). Notably, tumors from the ZIFOA‐M group exhibited significantly elevated levels of pro‐inflammatory cytokines (IL‐6, TNF‐α, and IFN‐γ), indicative of a robust immunostimulatory response (Figure 7e). Quantitative analysis of CD8^+^ T cells in lymph nodes and spleen demonstrated that the ZIFOA‐M treatment effectively stimulated immune responses (Figures S14 and S15). Furthermore, flow cytometry analysis revealed a marked increase in tumor‐infiltrating CD8^+^ T cells, coupled with a substantial reduction in immunosuppressive M2 macrophages and Tregs, suggesting that ZIFOA‐M effectively reprogrammed the TME from an immunosuppressive to an immunogenic state (Figure 7f–h). Moreover, the survival analysis further confirmed the superior anti‐tumor efficacy of ZIFOA‐M in the murine models (Figure S16). Collectively, these results demonstrated that ZIFOA‐M exerted potent antitumor effects by reversing immunosuppressive TME hallmarks, promoting cytotoxic T cell recruitment, and eliciting a pro‐inflammatory immune response.

To elucidate the therapeutic efficacy at the tissue level, tumors and multiple organs (including heart, liver, spleen, lungs, and kidneys) were collected from mice treated with different therapeutic regimens and performed comprehensive histopathological analyses. Tissue immunohistochemical staining of tumors further validated these findings, demonstrating enhanced CD8^+^ T cells infiltration and widespread tumor cells apoptosis in ZIFOA‐M treated mice. Critically, histopathological evaluation of the major organs confirmed the biosafety of ZIFOA‐M, with no evidence of treatment‐related toxicity (Figure S17). Moreover, the near absence of metastatic tumor nodules in lung underscored the system's ability to inhibit distal tumor dissemination (Figures S17 and S18). Comprehensively, our findings highlighted its dual functionality as both a tumor growth suppressor and a metastasis inhibitor, positioning it as a promising therapeutic candidate for breast cancer immunotherapy.

### 2.8 Inhibition of Tumor Recurrence

**FIGURE 8:**
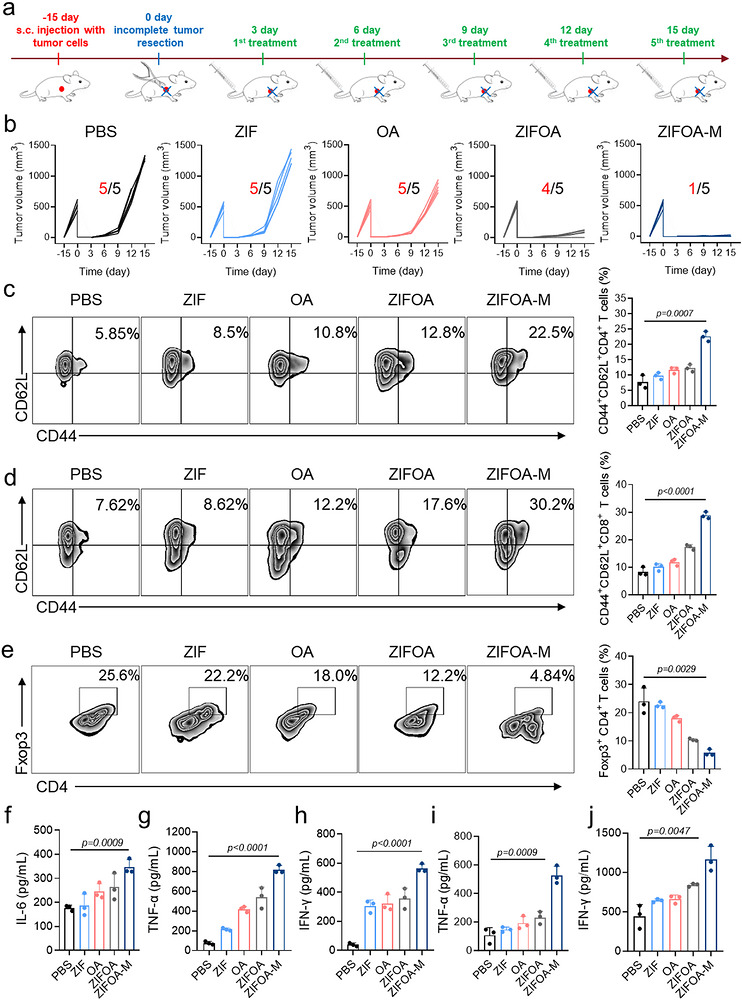
Inhibition of tumor recurrence and induction of long‐term immune memory. (a) Schematic of the tumor recurrence treatment. (b) Tumor recurrence rates post‐treatment. (c,d) Flow cytometry analysis of memory CD4^+^ and CD8^+^ T cells (CD44^+^CD62L^+^) in spleen. (e) Flow cytometry analysis of Tregs (CD4^+^Foxp3^+^). (f–h) Evaluation of pro‐inflammatory cytokines (TNF‐α, IFN‐γ, and IL‐6) in tumors. (i,j) Serum cytokine levels of TNF‐α and IFN‐γ. Data were expressed as mean ± standard deviations (SD) (*n* = 5).

To investigate whether ZIFOA‐M could induce immune memory, we established a tumor recurrence model. Mice with established tumors underwent surgical removal of the tumors and were subsequently treated with different formulations every 3 days for a total of 5 treatments (Figure 8a). As shown in Figure 8b, the ZIFOA‐M treatment group exhibited the lowest tumor recurrence rate in mice. Furthermore, flow cytometry analysis revealed that memory CD4^+^ and CD8^+^ T (CD44^+^CD62L^+^) cells in the spleen were increased by approximately 4‐fold (Figure 8c,d). Meanwhile, the proportion of Tregs in the ZIFOA‐M treated group was 5.6‐fold lower compared to the PBS control group and 5.35‐fold higher in the proportion of IFN‐γ^+^CD8^+^ T cells (Figure 8e and Figure S19), suggesting a significant reduction in immunosuppressive cells infiltration in the tumor. To evaluate the immunomodulatory effects, tumor lysates were analyzed via ELISA, revealing markedly elevated levels of pro‐inflammatory cytokines, including TNF‐α, IFN‐γ, and IL‐6 in the ZIFOA‐M group compared to other formulations (Figure 8f–h). Consistent with these findings, serum cytokine profiling demonstrated a systemic increase in TNF‐α and IFN‐γ (Figure 8i,j), indicating robust activation of systemic inflammatory responses.

## 3 Discussion

Macrophages are promising living carriers for tumor‐targeted drug delivery. Yet, existing research primarily utilizes them as simple transport systems. A primary limitation of this approach is the immunosuppressive tumor microenvironment, which restricts efficacy and neglects the cells’ own immunoregulatory potential. We used a bioactive oncolytic virus, rather than standard chemotherapeutics or nucleic acids, as the macrophage‐delivered payload. This choice was made to circumvent the functional limitations imposed by the immunosuppressive tumor microenvironment. The virus's multidimensional regulatory effects on tumor cells are key to breaking this constraint and unleashing macrophage potential.

Herein, a novel armored macrophage system of ZIFOA‐M was developed by bioorthogonally conjugating oncolytic adenovirus (OA)‐loaded ZIF‐8 onto the surface of M1 macrophages. This platform fundamentally redefines the role of macrophages in drug delivery. It has synergistically integrated inefficient in vivo targeting, OA‐induced immunomodulation, and suboptimal activation of adaptive immunity. Utilizing a bioorthogonal conjugation strategy, OA‐loaded particles were stably attached to macrophage surfaces without impairing their intrinsic functions, such as migration and surface marker expression. More importantly, the release of OA from ZIF‐8 in the acidic tumor microenvironment facilitated localized and sustained viral activity, avoiding the rapid systemic clearance seen with free OA. This targeting mechanism effectively overcomes a major limitation of off‐target effects in oncolytic virus therapy. The more important findings were the OA‐mediated downregulation of key “don't eat me” signals, CD47 and CD24, on tumor cells to 41.9% and 35.5% of baseline levels, respectively. Meanwhile, the OA‐induced immunogenic cell death (ICD) created a highly immunogenic tumor milieu. Consequently, ZIFOA‐M macrophages exhibited a drastically enhanced phagocytic capacity (66.6% vs. 2.4% for naïve M1), transforming them into efficient antigen‐presenting cells.

Additionally, ZIFOA‐M orchestrated a durable and self‐amplifying immune response. The platform not only initiated innate phagocytosis but also ensured its sustenance by maintaining a stable M1‐polarized state within the immunosuppressive TME. Along with the treatment, ZIFOA‐M macrophages retained high expression of M1 markers (CD80/CD86) and pro‐inflammatory cytokines (TNF‐α, IFN‐γ). Furthermore, the enhanced antigen presentation by ZIFOA‐M macrophages led to robust activation and proliferation of antigen‐specific CD8^+^ T cells, demonstrates significant reprogramming of the TME from “cold” to “hot.” The in vivo efficacy of this multi‐pronged approach was unequivocal. In an orthotopic 4T1 breast cancer model, ZIFOA‐M treatment achieved an 87.2% tumor suppression rate, significantly outperforming all control groups.

Our research aligns with the emerging field of cell engineering therapies and has achieved significant progress based on this foundation. Previous studies typically combined macrophages with checkpoint inhibitors or cytokines, but they often only addressed a single obstacle. However, the ZIFOA‐M platform represents a fundamental shift, as it can collaboratively solve problems such as blocked phagocytosis (through the downregulation of CD47/CD24), unstable polarization (through ICD and continuous signal transduction triggered by OA), and adaptive immune activation (through enhanced antigen cross‐presentation). All of these are achieved within a single entity targeting the tumor. Using bioactive oncolytic viruses as the core immunomodulator, rather than small molecules or antibodies, provides a dynamic, self‐replicating, and multifunctional stimulation, which is particularly suitable for synergistic action with living cell vectors.

Future studies should evaluate the efficacy of this platform in humanized mouse models or in human monocyte‐derived macrophages for a broader range of human tumors to enhance its clinical relevance. The potential of ZIFOA‐M can be further enhanced through combinatorial strategies. Given its role in constructing an immunogenic tumor microenvironment, combining ZIFOA‐M with immune checkpoint inhibitors is a reasonable next step to fully unleash the activated T‐cell response. Finally, exploring the genetic engineering of macrophages to express other immunomodulators (such as cytokines, bispecific linkers) in combination with ZIFOA may create a more powerful and customizable “living drug factory.”

## 4 Conclusions

In summary, we developed ZIFOA‐M as an OA‐armed macrophage platform that synergistically integrated oncolytic virotherapy with M1 macrophage engineering to overcome the key limitations of conventional macrophage‐based immunotherapy. By conjugating OA‐loaded ZIF‐8 to macrophage membranes via bioorthogonal chemistry, ZIFOA‐M achieves: (1) pH‐responsive viral release to locally disrupt “don't eat me” signals (CD47/CD24), enhancing phagocytic capacity; (2) sustained M1 polarization through OA‐induced ICD; and (3) robust adaptive immune activation via antigen cross‐presentation and CD8^+^ T cells priming. In murine breast cancer models, ZIFOA‐M mediated superior tumor suppression, significantly reduced CD47/CD24 expression, and expanding of antigen‐specific T cell populations without inducing systemic toxicity. This study establishes a blueprint for next‐generation macrophage therapies that coordinate innate phagocytosis, immunomodulation, and adaptive immunity, offering a clinically translatable strategy for solid tumor immunotherapy. Future investigations will explore ZIFOA‐M in combination with immune checkpoint inhibitors and its efficacy across diverse tumor models.

## 5 Experimental Section

### 5.1 Animals

Female Balb/c mice (7–8 weeks old) were obtained from Beijing Vital River Laboratory Animal Technology Co., Ltd. All animal procedures were conducted in strict compliance with the Guide for the Care and Use of Laboratory Animals. The experimental protocol was reviewed and approved by the Institutional Animal Care and Use Committee (IACUC) of the Wenzhou Institute, University of Chinese Academy of Sciences (Ethical Approval Nos. WIUCAS25061903).

### 5.2 Virus Titer of OA

The OA and OA‐GFP were generously provided by Fubio Biotechnology Co., Ltd. The viral titer of OA was determined using a modified protocol based on established methods [48, 49]. In brief, 293T cells were seeded in 96‐well plates (Guangzhou Jet Bio‐Filtration Co., Ltd.) at a density of 3000 cells per well and allowed to adhere overnight. The cells were then infected with OA and incubated for 72 h. Notably, the viral titer was quantified by measuring GFP fluorescence intensity for enhanced precision and efficiency.

### 5.3 Preparation and Characterization of ZIFOA

*ZIF_NH2_ synthesis*: A solution containing 2‐aminobenzimidazole (0.53 g, MACKLIN), 2‐methylimidazole (0.5 g, MACKLIN), and zinc nitrate hexahydrate (0.37 g, CHRON CHEMICALS) in methanol (50 mL, Aladdin) was vigorously stirred for 1.5 h at room temperature to yield aminated ZIF‐8 (ZIF_NH2_). *DBCO conjugation*: The obtained ZIF_NH2_ was subsequently functionalized with DBCO‐NHS ester through continuous stirring at 37°C for 2 h, resulting in ZIF_DBCO_. *OA loading*: For OA encapsulation, 10 mg of ZIF_DBCO_ was incubated with 1 × 10^8^ GFU of OA in an ice bath for 6 h to prepare the ZIFOA complex.

### 5.4 Macrophage Conjugation

Glycosylated macrophages were then incubated with ZIFOA (512 µg/mL) for 1 h to obtain the final ZIFOA‐M complex. *Size and zeta potential analysis*: Samples (ZIFOA, ZIF_DBCO_, and ZIF_NH2_) were appropriately diluted in PBS and analyzed using dynamic light scattering (DLS, ZEN 3600 Zetasizer, Malvern) for particle size distribution and surface charge determination.

### 5.5 pH‐Responsive Stability

The acid stability of ZIFOA was evaluated in a tumor‐mimicking acidic environment (pH 6.5, adjusted using 1 M HCl/NaOH). After dissolving 10 mg ZIFOA in 1 mL acidic solution, particle size changes were monitored at 4, 8, 12, and 24 h intervals using particle tracking analysis.

### 5.6 SEM Imaging

Sample aliquots (100 µL) were deposited on silicon wafers, vacuum‐dried, and gold‐sputtered (120 s) prior to field‐emission scanning electron microscopy (SEM, SU8010, HITACHI) observation.

### 5.7 TEM Analysis

OA samples were fixed in 2.5% glutaraldehyde (PBS, overnight) followed by 1% osmium tetroxide (1 h). After uranyl acetate staining (2%, aqueous), samples underwent graded ethanol dehydration (50%–100%) and acetone treatment before epoxy resin embedding. Ultrathin sections (10 nm) were examined by transmission electron microscopy (TEM, Talos F200S G2, FEI).

### 5.8 Fourier Transform Infrared Spectroscopy (FTIR) Determination

The ZIF_NH2_ powder was thoroughly mixed with KBr powder, ground uniformly, and pressed into pellets. These pellets were analyzed using FTIR (Tensor II, Bruker) with a detection range of 4000 to 400 cm^−^^1^. The measurement settings included a frequency of 10 kHz and a scanning time of 16 s.

### 5.9 Preparation and Characterization of ZIFOA‐M

RAW264.7 macrophages were obtained from the Cell Resource Center of the Shanghai Institute of Biological Sciences, Chinese Academy of Sciences. The cells were cultured in high‐glucose Dulbecco's Modified Eagle's Medium (DMEM, MeilunBio, China) supplemented with 10% fetal bovine serum (FBS), 100 U/mL penicillin, and 100 mg/mL streptomycin, under a humidified atmosphere of 5% CO_2_ at 37°C. To polarize macrophages to the M1 phenotype, cells were treated with 100 ng/mL lipopolysaccharide (LPS, Novoprotein, China) and 20 ng/mL IFN‐γ (Novoprotein, China) for 24 h [50]. Subsequently, M1 macrophages were incubated with 10 µM Ac4ManNAz (MACKLIN) for 3 days to achieve surface glycosylation.

For fluorescence labeling, 1 × 10^8^ GFU of OA was conjugated with 10 µM Cy5‐NHS (MCE, USA) at 37°C for 2 h, followed by centrifugation to purify Cy5‐labeled OA (OA‐Cy5). ZIFOA, prepared from OA‐Cy5, was co‐incubated with M1 macrophages to generate Cy5‐labeled ZIFOA‐M. Unbound ZIFOA was removed by washing with PBS, and the cells were fixed with 4% paraformaldehyde (Beyotime, China) for 30 min. After additional PBS washes, cells were permeabilized with 1% Triton X‐100 (Biofroxx, Germany) for 30 min, followed by three PBS washes. Cells were then blocked with 1% BSA for 1 h. After three further PBS washes, a green fluorescent probe for microfilaments (Beyotime, China) was diluted 1: 100, and 30 µL of diluted phalloidin was added to each sample. To prevent drying, water was added around the samples, and staining was performed overnight. Following three PBS washes, 5 µL of DAPI‐containing anti‐quenching mounting medium (Beyotime, China) was applied to the slides. Coverslips were inverted onto the mounting medium and sealed with nail polish. Co‐localization of macrophages and Cy5 was visualized using confocal laser scanning microscopy (CLSM, A1, Nikon).

Macrophages were incubated in serum‐free medium for 12 h. M and ZIFOA‐M cells were seeded in the upper chamber of a Transwell insert, with serum‐containing medium added to the lower chamber. Following a 6 h migration period, the insert was removed and fixed in 4% paraformaldehyde for 30 min. The cells within the insert were then stained with crystal violet and observed and quantified under an optical microscope.

### 5.10 Determination of Cell Surface Proteins and Virus Viability

M and ZIFOA‐M cells were incubated with 1% bovine serum albumin (BSA) in phosphate‐buffered saline (PBS) for 10 min at room temperature to block nonspecific binding. Following PBS washing to remove excess BSA, cell surface markers were stained using the following fluorochrome‐conjugated antibodies: phycoerythrin (PE)‐labeled anti‐F4/80 (clone BM8; BD Pharmingen, San Diego, CA, USA) and allophycocyanin (APC)‐conjugated anti‐CD11b (clone M1/70; BD Pharmingen).

After 30 min of incubation at 4°C in the dark, cells were washed once with PBS to remove unbound antibodies and resuspended in 200 µL of fresh PBS. Flow cytometric analysis was immediately performed using a CytoFLEX flow cytometer (Beckman Coulter, Brea, CA, USA) equipped with 488‐nm and 640‐nm lasers. Data acquisition and analysis were conducted using CytExpert software (v2.4; Beckman Coulter), with appropriate isotype controls and fluorescence minus one (FMO) control included for proper gating strategy.

293T cells were cultured in 24‐well plates at an initial seeding density of 5 × 10^4^ cells per well. Following cell attachment, the cells were treated with OA, ZIFOA, and ZIFOA‐M formulations containing GFP reporter (with a standardized dose of 2.5 × 10^4^ GFP units of OA‐GFP). After 24 h of incubation, cellular infection efficiency was quantitatively assessed by fluorescence microscopy, with GFP‐positive cells serving as an indicator of viral infectivity and biological activity. This experimental approach allowed for direct visualization and comparative evaluation of the infection capabilities among the different OA formulations.

### 5.11 Analysis of Macrophage Polarization and Phagocytic ability

M, ZIF‐M, and ZIFOA‐M derived from RAW264.7 cells were seeded in 24‐well plates at a density of 5 × 10^4^ cells per well and co‐cultured with or without an equal number of 4T1 cells. To assess gene expression, total RNA was extracted using an RNA extraction kit (Sangon Biotech, China) and reverse‐transcribed into cDNA with a reverse transcriptase kit (Takara, Japan). Quantitative real‐time PCR (qRT‐PCR) was conducted using TB Green PCR Master Mix (Takara, Japan), with glyceraldehyde‐3‐phosphate dehydrogenase (GAPDH) serving as the internal reference. Relative gene expression levels were quantified using the 2^−ΔΔ^ CT method. Primer sequences for qRT‐PCR are provided in Table S1.

Supernatants were collected, and concentrations of IFN‐γ, IL‐1β, IL‐6, and TNF‐α were measured using ELISA kits (Peprotech, USA). Flow cytometry was employed to evaluate macrophage phenotypic changes. Cells were blocked with PBS containing 1% BSA at 4°C for 10 min, followed by surface staining with PE‐ or PC5.5‐conjugated F4/80, FITC‐conjugated CD86, PC5.5‐conjugated CD80, or PE‐conjugated CD163 antibodies (BD Pharmingen, USA) at 4°C for 30 min. For intracellular staining, cells were fixed and permeabilized using a fixation/permeabilization kit (BD Pharmingen, USA) and stained with APC‐conjugated CD206 (BD Pharmingen, USA). Cells were then washed with PBS containing 1% FBS and analyzed using a flow cytometer.

### 5.12 Analysis of Macrophage Phagocytic Ability

The 4T1‐GFP mouse breast cancer cell line, labeled with green fluorescent protein, was uniformly seeded in confocal dishes at a density of 5 × 10^4^ cells per well. The following day, after most tumor cells had adhered to the dish surface, macrophages were stained with a red fluorescent probe targeting the cell membrane (DiD, MCE, USA). Subsequently, macrophages (M, ZIFOA‐M, or ZIFOA‐M) were added at the same density for co‐culture over 24 h, with an OA concentration of 2.5 × 10^4^ GFU. Phagocytosis of tumor cells by macrophages was visualized using confocal laser scanning microscopy. Tumor cells were observed at 488 nm (green), macrophages at 640 nm (red), and cell nuclei at 405 nm (blue).

### 5.13 Cell Cytotoxicity with Different OA Formulations

4T1 cells were seeded in 96‐well plates at a density of 5000 cells per well and allowed to adhere overnight. Subsequently, the cells were treated with ZIFOA‐M, ZIFOA, and OA at varying modification ratios, along with the corresponding control groups (ZIF and PBS). After 24 h of co‐incubation, 100 µL of CCK‐8 solution (4 mg/mL, Dojindo, Kumamoto, Japan) was added to each well, followed by incubation at 37°C for 4 h in a humidified atmosphere containing 5% CO_2_. The optical density (OD) at 450 nm was measured using a microplate reader (ELx800, BioTek, USA). Cell viability was calculated according to the manufacturer's protocol.

### 5.14 Detection of the “Don't Eat Me” Signals

4T1 cells were seeded in 24‐well plates at a density of 5 × 10^4^ cells per well and treated with ZIFOA‐M, ZIFOA, OA, ZIF, or PBS (each containing an equivalent OA dose of 2.5 × 10^4^ GFU) for 24 h. After incubation, the cells were harvested, blocked with PBS containing 1% BSA at 4°C for 10 min, and subsequently stained with APC‐conjugated anti‐CD47 (BD Pharmingen, USA) and PE‐conjugated anti‐CD24 (BD Pharmingen, USA) at 4°C for 30 min in the dark. Following surface staining, the cells were washed twice with PBS supplemented with 1% FBS and analyzed by flow cytometry (BD FACSCanto II).

### 5.15 Cell Cytotoxicity of ZIFOA‐M

The cytotoxicity of OA, ZIFOA, ZIFOA‐M, ZIF, and PBS in L929, RAW264.7, and 4T1 cells was evaluated using the CCK‐8 assay. Briefly, cells were seeded in 96‐well plates at a density of 5000 cells per well and incubated overnight to allow adherence. After treatment with the respective compounds (with OA at a concentration of 2500 GFU), the cells were further cultured for the specified time periods. Subsequently, 100 µL of CCK‐8 solution (4 mg/mL) was added to each well, followed by incubation at 37°C for 4 h. The optical density (OD) at 450 nm was measured using a microplate reader, and cell viability was calculated according to the manufacturer's protocol. Each experiment was performed in triplicate to ensure reproducibility.

### 5.16 Immunogenic Cell Death Assay

4T1 cells were seeded in 24‐well plates at a density of 5 × 10^4^ cells per well and treated with ZIFOA‐M, ZIFOA, OA, ZIF, or PBS (containing 2.5 × 10^4^ GFU of OA) for 24 h. After co‐culture, the cells were fixed with 4% paraformaldehyde for 30 min, washed with PBS, and blocked with 1% goat serum at room temperature for 1 h. Subsequently, cells were permeabilized with 1% Triton X‐100 for 30 min, followed by incubation with primary antibodies against HMGB1 or CRT (Bioss, China) at 4°C overnight. After PBS washing, cells were incubated with a 488 nm‐conjugated secondary antibody (Cohesion Biosciences, UK) in the dark for 1 h. Finally, the samples were mounted, and fluorescence signals were visualized using a confocal microscope. For ATP release analysis, cell supernatants were collected, and extracellular ATP levels were quantified using a commercial ATP detection kit (Beyotime, China) following the manufacturer's instructions.

### 5.17 Cell Apoptosis Detection

4T1 cells were seeded in 24‐well plates at a density of 5 × 10^4^ cells per well and incubated with ZIFOA‐M, ZIFOA, OA, ZIF, or PBS (containing an equivalent OA dose of 2.5 × 10^4^ GFU) for 24 h. After treatment, cell apoptosis was assessed using an Annexin V‐APC/PI apoptosis detection kit (eBioscience, USA) according to the manufacturer's protocol, followed by flow cytometric analysis (BD FACSCanto II).

### 5.18 Neutralization Assay

The 293T cells were seeded in 24‐well plates at a density of 5 × 10^4^ cells per well and allowed to adhere overnight. The AAV9 neutralizing antibody (FUBIO, China) was serially diluted at a 1: 2 ratio to achieve a final dilution of 1/1024. Subsequently, OA, ZIFOA, and ZIFOA‐M (each containing 2.5 × 10^4^ genome‐forming units (GFU) of OA) were pre‐incubated with the diluted neutralizing antibodies at varying concentrations for 1 h at 37°C. Following incubation, the antibody‐treated mixtures were added to the 293T cells and co‐cultured for an additional 24 h under standard conditions. After incubation, the cells were first examined under a fluorescence microscope to assess transduction efficiency, then harvested and subjected to flow cytometry for quantitative fluorescence analysis to determine the neutralizing antibody's inhibitory effect on AAV9‐mediated transduction.

### 5.19 Pharmacokinetic Analysis and Virus Distribution Assay

For pharmacokinetic analysis, Balb/c mice were intravenously administered with ZIFOA‐M, ZIFOA, or OA (10^7^ GFU dose), and whole blood samples were collected at predetermined time points. Genomic DNA was extracted from the whole blood using a commercial blood genomic DNA extraction kit (TIANGEN, China) following the manufacturer's protocol. The E1A gene copy number in peripheral blood was then quantified by qPCR using specific primers (sequences provided in Table S1).

Cy5‐labeled oncolytic adenovirus (OA), ZIFOA, and ZIFOA‐M (1 × 10^7^ GFU per mouse) were intravenously administered to Balb/c mice via tail vein injection. After 4 h of circulation, the mice were euthanized, and tumors along with major organs were harvested for ex vivo fluorescence imaging. The biodistribution of Cy5‐labeled OA in different tissues was quantitatively analyzed using a multimodal in vivo imaging system (IVIS Lumina XRMS Series III, PerkinElmer) to evaluate the tumor‐targeting efficiency of each formulation.

### 5.20 In Vivo Tumor Cellular Uptake of Different Formulations

To evaluate tumor targeting efficiency, tumor‐bearing mice were intravenously injected with ZIFOA‐M, ZIFOA, or OA (10^7^ GFU of OA‐Cy5) via the tail vein. After 12 h, the tumor tissues were excised, rinsed with 1 × PBS to remove surface blood, and transferred into 5 mL centrifuge tubes. The tissues were mechanically dissociated using tweezers and enzymatically digested in 4 mL of digestion cocktail containing 1 mg/mL collagenase IV, hyaluronidase, and 100 µg/mL DNase (Biosharp, China) at 37°C with continuous shaking at 180 × rpm for 1 h. The resulting cell suspension was filtered through a 100‐µm mesh sieve into a 15 mL centrifuge tube, followed by centrifugation at 450 × g for 5 min to pellet the cells. After discarding the supernatant, the cell pellet was treated with 2 mL of red blood cell lysis buffer (Biosharp, China) at 4°C for 10 min to remove erythrocytes. The lysate was then diluted with 1 × PBS and centrifuged at 800 ×g for 5 min to collect the cells, which were subsequently resuspended in 2 mL of 1 × PBS for cell counting. Finally, 10^5^ cells were subjected to flow cytometry analysis to quantify the proportion of OA‐Cy5‐positive tumor cells.

### 5.21 Virus Penetration of 3D Tumor Spheroids

To generate tumor spheroids, PBS containing agar was dispensed into 24‐well plates (200 µL/well) and allowed to solidify. Subsequently, 4T1‐mCherry cells were seeded at a density of 5 × 10^4^ cells/well, gently mixed for uniform distribution, and cultured at 37°C for 48 h to allow spheroid formation. The resulting multicellular spheroids were then transferred to confocal dishes and co‐cultured for 24 h with OA, ZIFOA, ZIFOA‐M, ZIF, or PBS (all containing 5 × 10^4^ GFU‐equivalent OA). Finally, the spheroids were subjected to confocal laser scanning microscopy for z‐stack imaging to obtain cross‐sectional fluorescence profiles at varying depths.

### 5.22 TissueFAXS Cytometry Panoramic Tissue Quantitative Experiment

To evaluate the cellular uptake and spatial interaction between 4T1‐GFP cells and macrophages, 4T1‐GFP cells were seeded at a density of 5 × 10^4^ cells per well on cell coverslips in 24‐well plates and co‐cultured with an equal quantity of DiD‐labeled ZIF‐M and ZIFOA‐M for 24 h. Following incubation, the cells were fixed with 4% paraformaldehyde and mounted for imaging. Fluorescence images of the specimens were acquired using the TissueFAXS spectral imaging system (TissueFAXS Plus, TissueGnostics), and spatial analysis was conducted with StrataQuest software (version 7.1.129, TissueGnostics GmbH, Vienna, Austria) to quantitatively assess the intercellular distances between 4T1‐GFP cells and macrophages.

### 5.23 In Vitro Proliferation Experiment of CD8^+^ T Cells

4T1 cells were seeded in 24‐well plates at a density of 5 × 10^4^ cells per well and treated with ZIFOA‐M, ZIFOA, OA, ZIF, or PBS (each containing 2.5 × 10^4^ GFU of OA) for 24 h. Subsequently, splenocytes isolated from Balb/c mice were plated in 24‐well plates at 5 × 10^5^ cells per well and co‐cultured for 72 h. After incubation, the cells were harvested and stained with fluorochrome‐conjugated antibodies, including APC‐anti‐CD3, PC5.5‐anti‐CD4, and PE‐anti‐CD8 (BD Pharmingen, USA), followed by a PBS wash to remove unbound antibodies. The stained cells were resuspended in 200 µL PBS and analyzed by flow cytometry to assess CD8^+^ T cell proliferation.

### 5.24 Anti‐Tumor and Recurrence via ZIFOA‐M In Vivo

To establish a breast cancer model, female Balb/c mice were intravenously injected with 1 × 10^6^ 4T1 cells. In the first experimental setup, after 15 days of tumor inoculation, the mice were randomly divided into treatment groups and administered intravenous injections of ZIFOA‐M, OA, ZIFOA, ZIF, or PBS (1 × 10^7^ GFU per dose) every three days for a total of five treatments, with tumor volume monitored daily to assess progression. In the second experimental setup, once the tumor volume reached 500–600 mm^3^, surgical resection was performed, followed by suturing. The mice were then randomized into treatment groups and received the same dosing regimen to evaluate tumor recurrence and progression, with daily measurements of tumor volume.

### 5.25 Immunohistochemistry and Immunofluorescence

The tissue specimens were fixed in 10% neutral buffered formalin and embedded in paraffin using standard protocols. Subsequently, 4‐µm‐thick sections were cut, dewaxed in xylene, and rehydrated through a graded ethanol series. For immunohistochemical (IHC) analysis, antigen retrieval was performed using citrate buffer (pH 6.0) at 95°C for 20 min, followed by blocking with 5% bovine serum albumin (BSA) for 1 h at room temperature. The sections were then incubated overnight at 4°C with primary anti‐CD8 antibody (1: 200 dilution; Cohesion Biosciences, UK), followed by appropriate secondary antibody detection. For apoptosis assessment, immunofluorescence staining was conducted using a TUNEL assay kit (Beyotime, China) according to the manufacturer's instructions. Finally, all stained sections were imaged and quantitatively analyzed using case‐analysis software, with appropriate controls included in each experiment.

### 5.26 Safety Evaluation

Following the final treatment, mice were euthanized for antitumor efficacy assessment, and tumor tissues along with major organs (heart, liver, spleen, lung, and kidney) were harvested, fixed in 4% paraformaldehyde (50 mL per sample), and processed for paraffin embedding. Tissue sections were prepared and stained with hematoxylin and eosin (H&E) using a commercial staining kit (Beyotime, China) according to the manufacturer's protocol. Histopathological examination was performed using an optical microscope (Carl Zeiss Axiovert 40CFL, Germany), and representative images were captured for subsequent analysis.

### 5.27 Statistical Analysis

Statistical analyses were performed using GraphPad Prism software (Version 8.0), with all quantitative data expressed as mean ± standard deviation (SD). Statistical significance was determined using two‐tailed unpaired Student's t‐test for comparison of two groups or by ANOVA for comparison of multiple groups, with a p‐value < 0.05 considered statistically significant; non‐significant results were denoted as “ns”. was evaluated with of variance.

### 5.28 Declaration of Generative AI and AI‐Assisted Technologies in the Writing Process

During the preparation of this work, the authors used Deepseek in order to proof the grammar and language. After using this tool/service, the authors reviewed and edited the content as needed and take full responsibility for the content of the publication.

## Author Contributions

J.W. and N.L. designed and performed the experiments. Y. C., L.Y., and S.Y. analysed the experimental data. Y.Z. established tumor models. J.W., N.L., L.Y., and J.D. helped with animal experiments. J.D. designed and synthesized the materials. Y.C., J.D. and N.L. wrote the manuscript. J.D. and Y.B. supervised the project, and all authors reviewed and edited the manuscript before submission.

## Conflicts of Interest

The authors declare no conflicts of interest.

## Supporting information




**Supporting File**: advs75406‐sup‐0001‐SuppMat.docx.

## Data Availability

The data that support the findings of this study are available from the corresponding author upon reasonable request.
