# Supplementary material for: Engineering Oncolytic Virus‐Armed Macrophages for Enhanced Cancer Immunotherapy
Source: Adv Sci (Weinh). 2026 Apr 20;13(40):e75406. doi: 10.1002/advs.75406 (PMC13335506; doi:10.1002/advs.75406)
Supplement: Supplementary file 1 — Supporting File: advs75406‐sup‐0001‐SuppMat.docx. [file ADVS-13-e75406-s001.docx]

**Engineering Oncolytic Virus-Armed Macrophages for Enhanced Cancer Immunotherapy**

Jilong Wang^a,b,^*^,#^, Ning Lu^a,b,d#^, Zhuo Yan^a,b^, Luyi Ye^a,b^, Luyao Bai^a,b^, Shanshan Yuan^a,b^, Yuting Zhu^a,b^, Yiru Xiong^a,b^, Yongheng Bai^a,c,^*, Yaping Chen^d,e,^*, Junjie Deng^a,b,^*

^a^ Joint Centre of Translational Medicine, The First Affiliated Hospital of Wenzhou Medical University, Wenzhou Medical University, Wenzhou, Zhejiang 325000, China

^b^ Joint Centre of Translational Medicine, Wenzhou Institute, University of Chinese Academy of Sciences, Wenzhou, Zhejiang 325000, China

^c^ Zhejiang Key Laboratory of Intelligent Cancer Biomarker Discovery and Translation, The First Affiliated Hospital, Wenzhou Medical University, Wenzhou, Zhejiang 325035, China

^d^ Oujiang Laboratory (Zhejiang Lab for Regenerative Medicine, Vision and Brain

Health), Wenzhou, Zhejiang 325000, China

^e^ Monash Institute of Pharmaceutical Sciences, Monash University, 381 Royal Parade, Parkville, VIC 3052, Australia

^#^ Jilong Wang and Ning Lu contributed equally to this work

* Corresponding authors: wangjilong@ucas.ac.cn (J. Wang), wzbyh@wmu.edu.cn (Y. Bai), [chenyaping@ojlab.ac.cn](mailto:chenyaping@ojlab.ac.cn) (Y. Chen), j.deng@ucas.ac.cn (J. Deng)

ORCiD ID: 0000-0002-0091-0333 (J. Wang), 0000-0003-3910-4731 (Y. Bai), 0000-0002-1940-0966 (Y. Chen), 0000-0002-5137-8615 (J. Deng)

**Table S1**. The sequences of primers used for qRT-PCR analysis

| E1A | Forward | 5' | CCTGAGACGCCCGACATC | 3' |
| --- | --- | --- | --- | --- |
|  | Reverse | 5' | GGACCGGAGTCACAGCTAT | 3' |
| IL-6 | Forward | 5' | CTGGAGCCCACCAAGAACGA | 3' |
|  | Reverse | 5' | GCCTCCGACTTGTGAAGTGGT | 3' |
| TNF-α | Forward | 5' | AGGGTCTGGGCCATAGAACT | 3' |
|  | Reverse | 5' | CCACCACGCTCTTCTGTCTAC | 3' |
| IL-1β | Forward | 5' | GCTGTGGAGAAGCTGTGGCA | 3' |
|  | Reverse | 5' | GGGAACGTCACACACCAGCA | 3' |
| GADPH | Forward | 5' | TGTGGATGGCCCCTCTGGAA | 3' |
|  | Reverse | 5' | TGACCTTGCCCACAGCCTTG | 3' |
| CD47 | Forward | 5' | gtccgtaatgtggaggcgca | 3' |
|  | Reverse | 5' | gccgtgcggtttttcagctc | 3' |
| CD24 | Forward | 5' | gcagatctccacgcaccgaa | 3' |
|  | Reverse | 5' | agcagtgccagaagcagcaa | 3' |


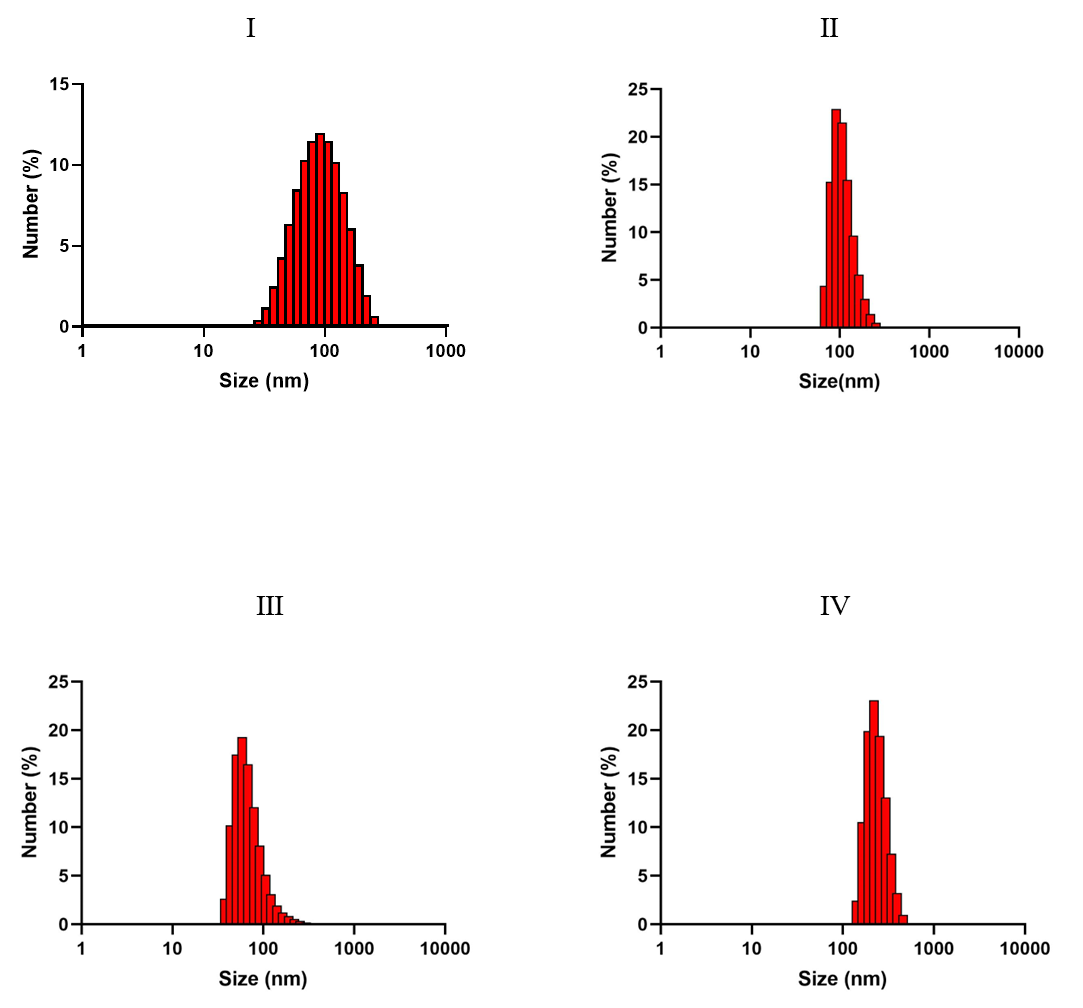


Figure S1. Particle size distribution of ZIF-NH₂ synthesized under different molar ratios of diaminobenzimidazole to dimethylimidazole. Ⅰ:no diaminobenzimidazole; Ⅱ: 1:4; Ⅲ: 3:7; Ⅳ: 2:3.


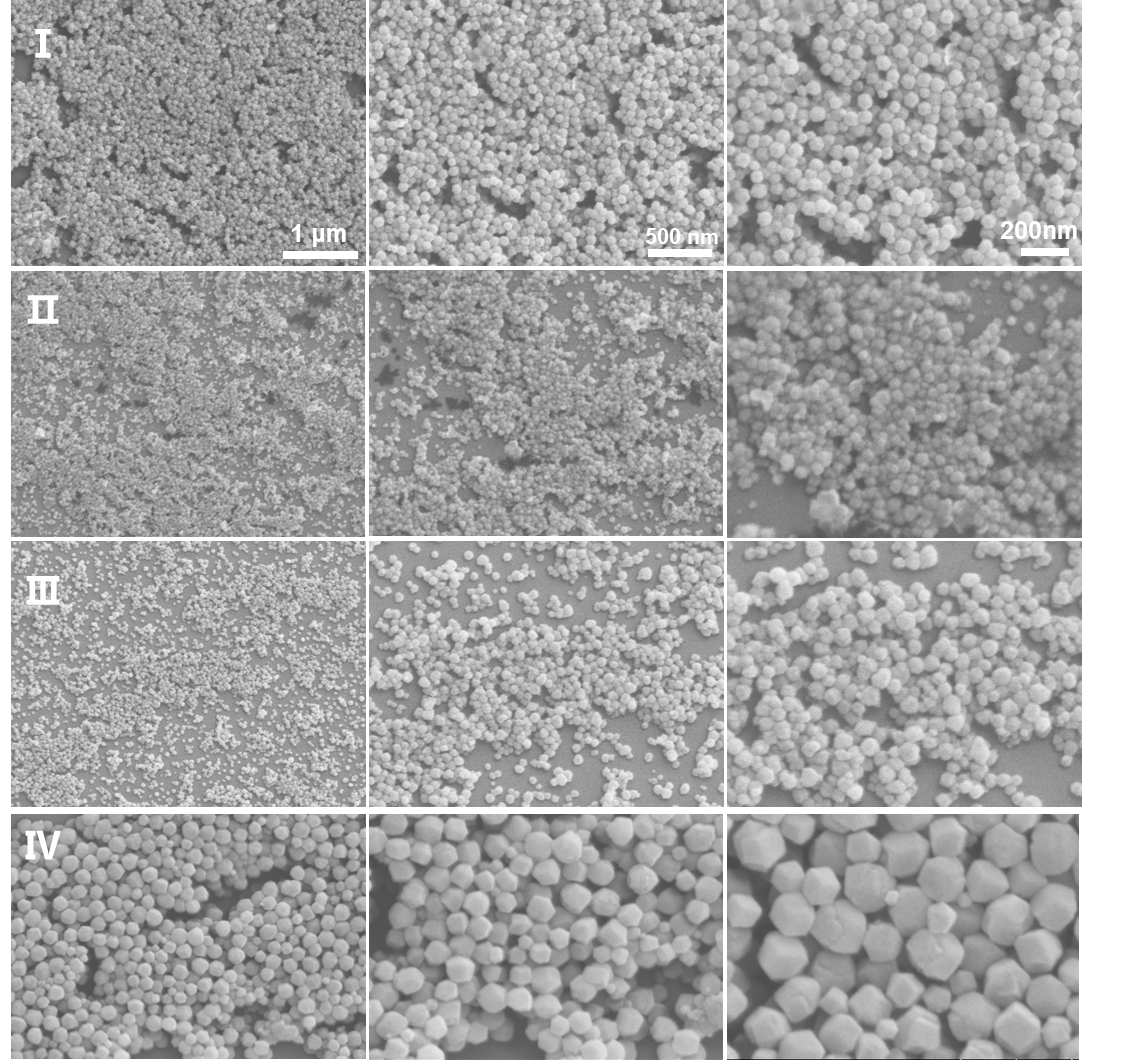


Figure S2. SEM diagram of ZIF-NH₂ synthesized under different molar ratios of diaminobenzimidazole to dimethylimidazole. Ⅰ: no diaminobenzimidazole; Ⅱ: 1:4; Ⅲ: 3:7; Ⅳ: 2:3.


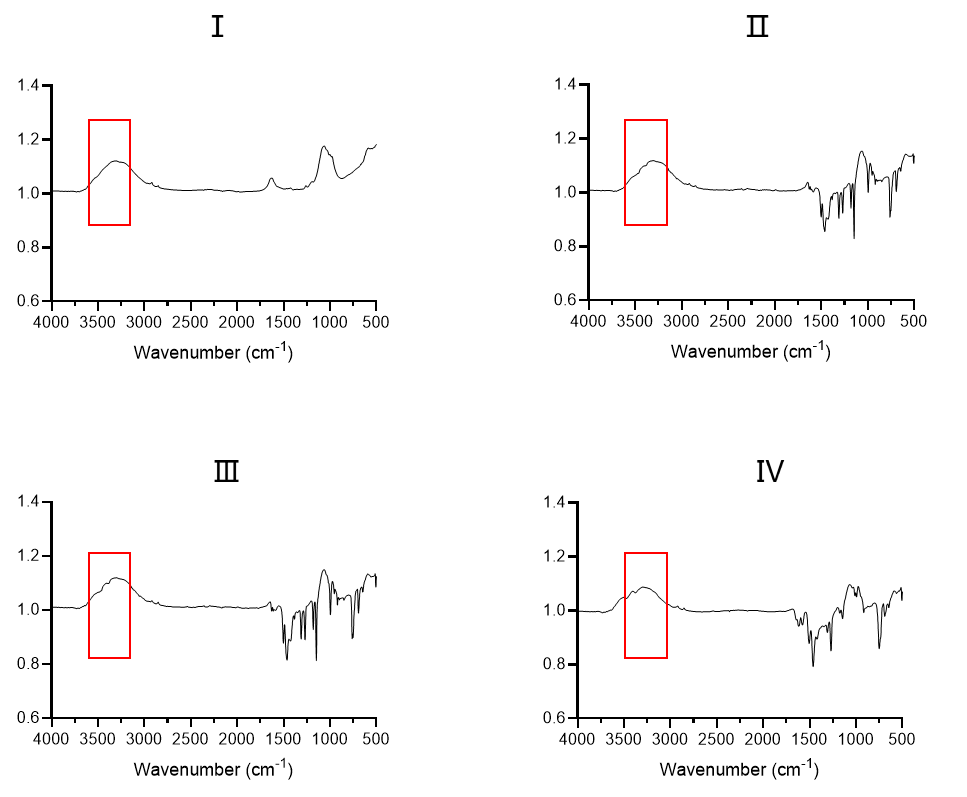


Figure S3. Fourier Transform Infrared Spectroscopy (FTIR) spectrum diagram of ZIF-NH₂ synthesized under different molar ratios of diaminobenzimidazole to dimethylimidazole. Ⅰ: no diaminobenzimidazole; Ⅱ: 1:4; Ⅲ: 3:7; Ⅳ: 2:3.

Figure S4. In vitro release profile of OA from ZIFOA in PBS of pH 7.4 and 6.5 at 37 ℃. Data were expressed as mean ± standard deviations (SD) (n = 3).


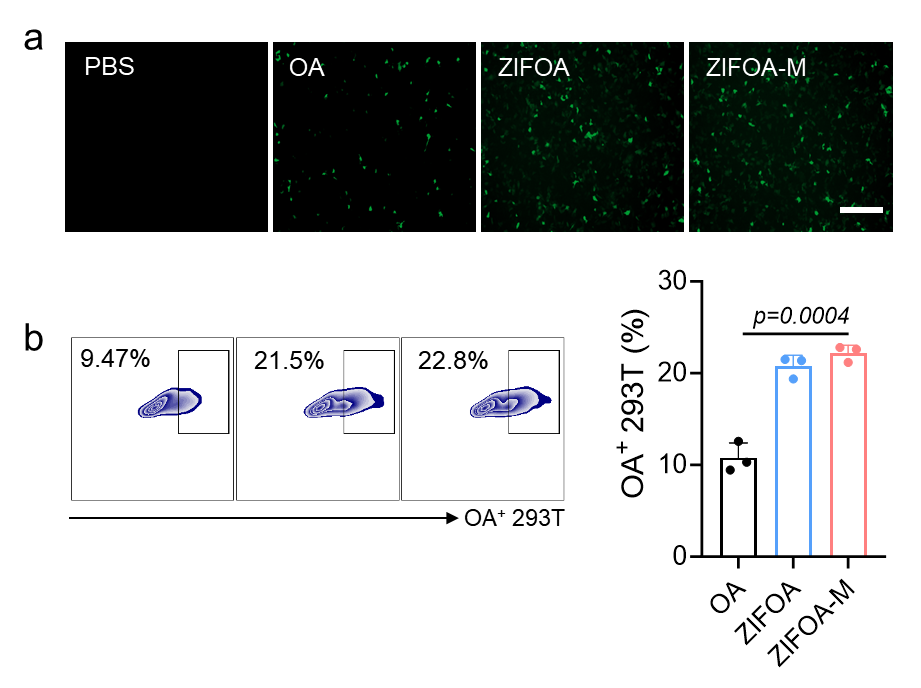


Figure S5. (a) Confocal microscopy images and (b) flow analysis of the anti-neutralizing ability of OA, ZIFOA and ZIFOA-M under another low concentrations of neutralizing antibodies *in vitro*. Scale bar: 100 μm. Data were expressed as mean ± standard deviations (SD) (n = 3).


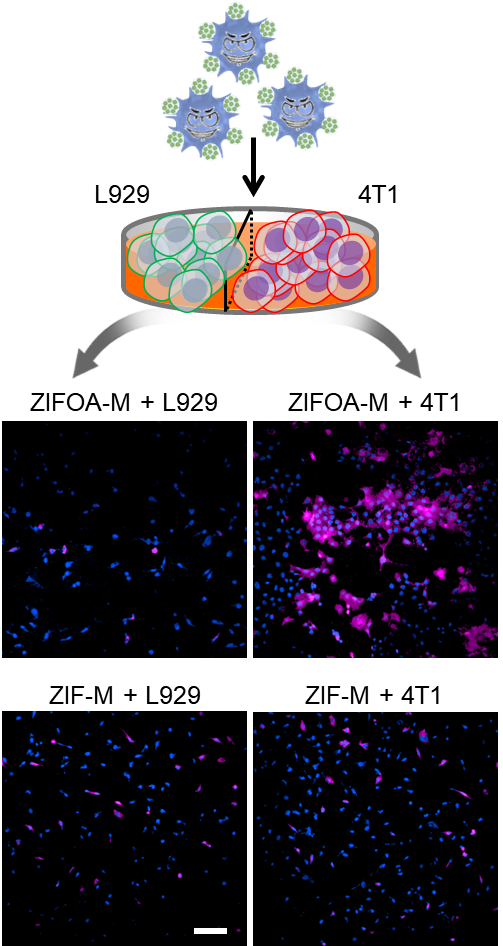


Figure S6. Tumor-targeting capability of ZIFOA-M between the L929 and 4T1 cells that they were co-cultured in distinct regions of the same well *in vitro*. Scale bar: 100 μm.


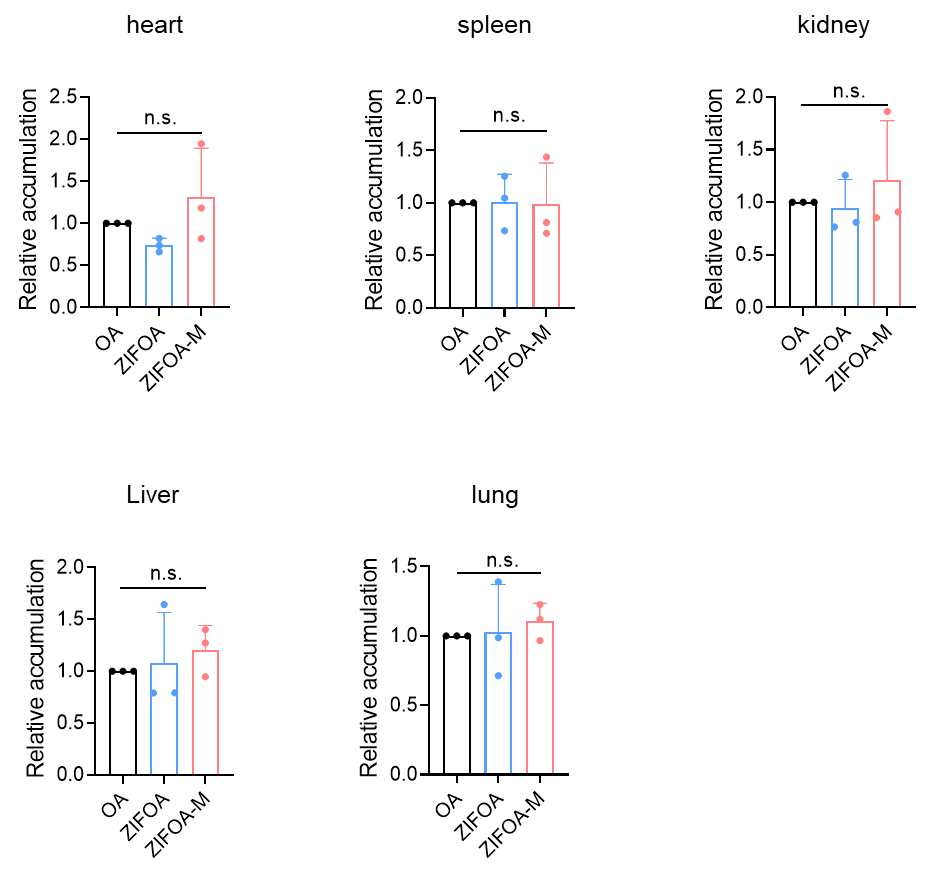


Figure S7. Fluorescence quantification of hearts, spleen and kidney after 4 hours post-injection of different OA derived formulations. Data were expressed as mean ± standard deviations (SD) (n = 3). n.s.= no significance.


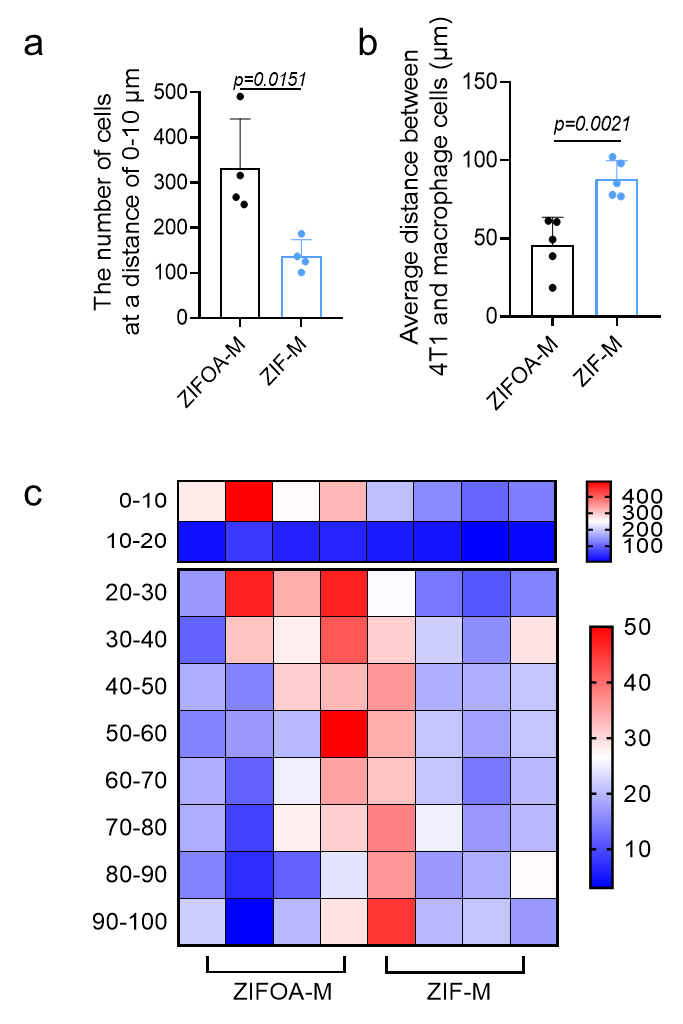


Figure S8. Tissue flow cytometry quantification of macrophages around the tumor cells in 10 μm (a). The average distance between tumor cells and macrophages (b). The hot map of whole distance and cell numbers distribution in the ZIFOA-M and ZIF-M groups. Data were expressed as mean ± standard deviations (SD) (n = 5).


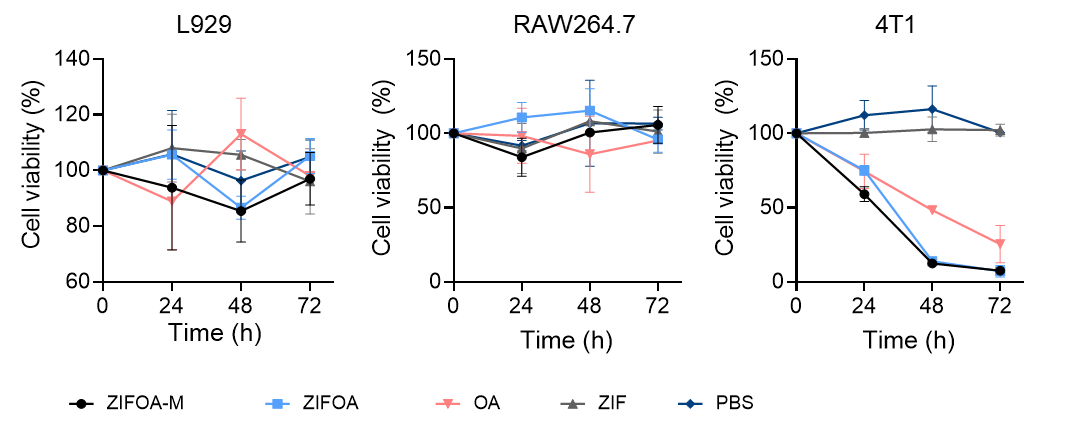


Figure S9. Cell cytotoxicity of ZIFOA-M, ZIFOA, OA, ZIF, and PBS against the normal and tumor cell lines (L929, RAW264.7 and 4T1). Data were expressed as mean ± standard deviations (SD) (n = 3).


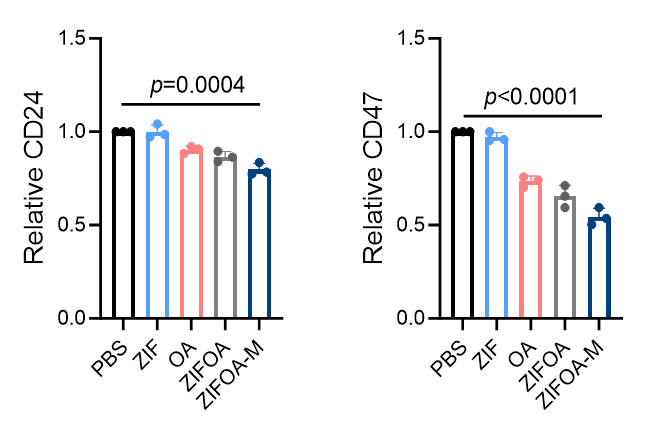


Figure S10. qPCR analysis of CD24/CD47 signaling pathway regulation on tumor cell surface by different OA formulations. Data were expressed as mean ± standard deviations (SD) (n = 3).


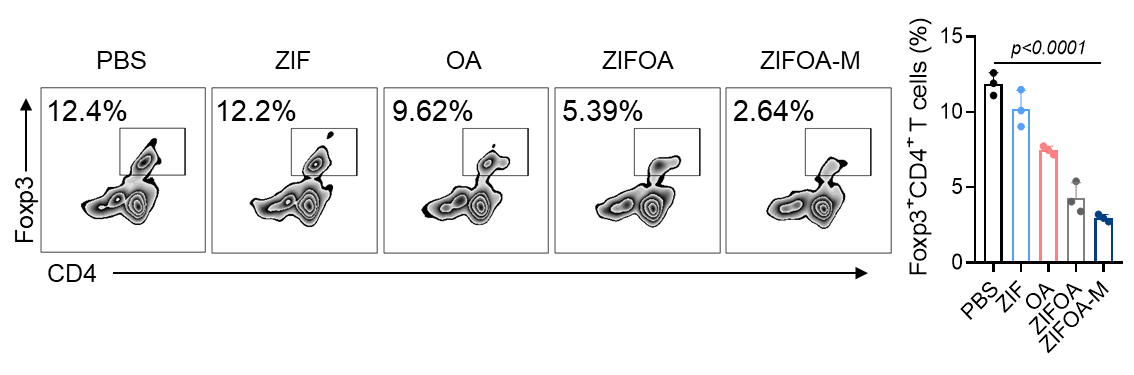


Figure S11. Flow cytometry analysis and statistical results of the *in vitro* suppression of Treg cells by ZIFOA-M, ZIFOA, OA, ZIF, and PBS. Data were expressed as mean ± standard deviations (SD) (n = 3).

Figure S12. The tumor inhibition rate among the mice treated by different formulations. Data were expressed as mean ± standard deviations (SD) (n = 5).

Figure S13. The body weight of mice during the treatment. Data were expressed as mean ± standard deviations (SD) (n = 5).


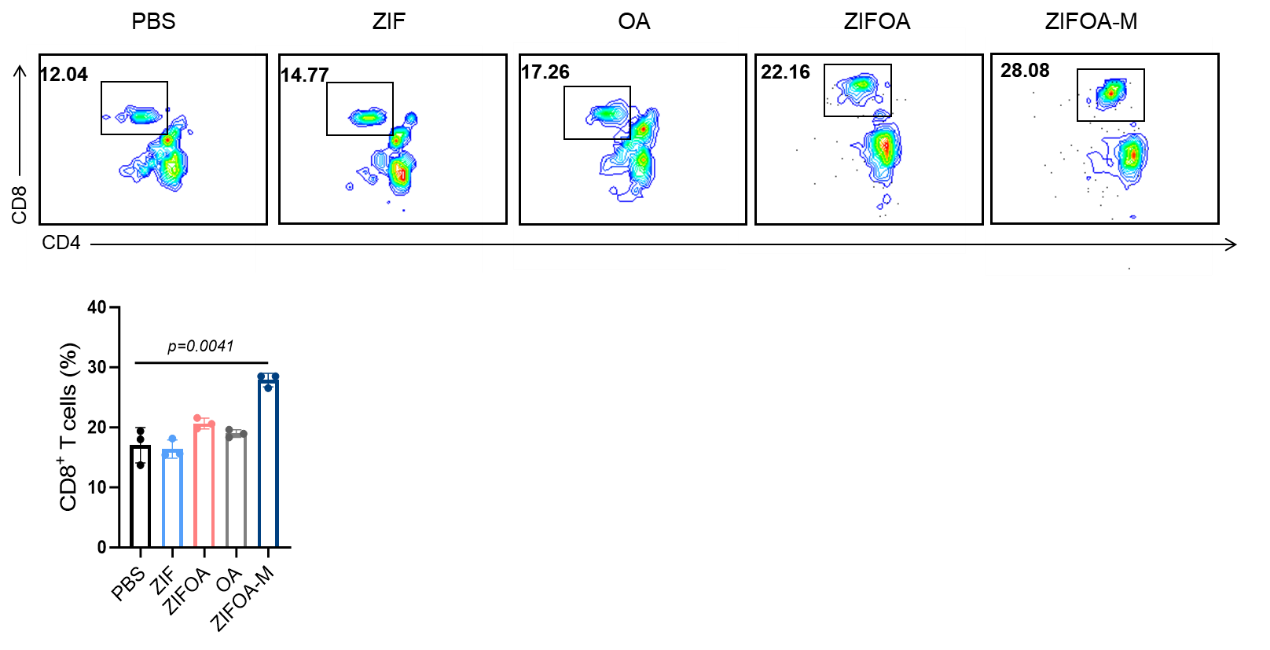


Figure S14. Quantitative analysis of CD8^+^ T cells in lymph nodes. Data were expressed as mean ± standard deviations (SD) (n = 3).


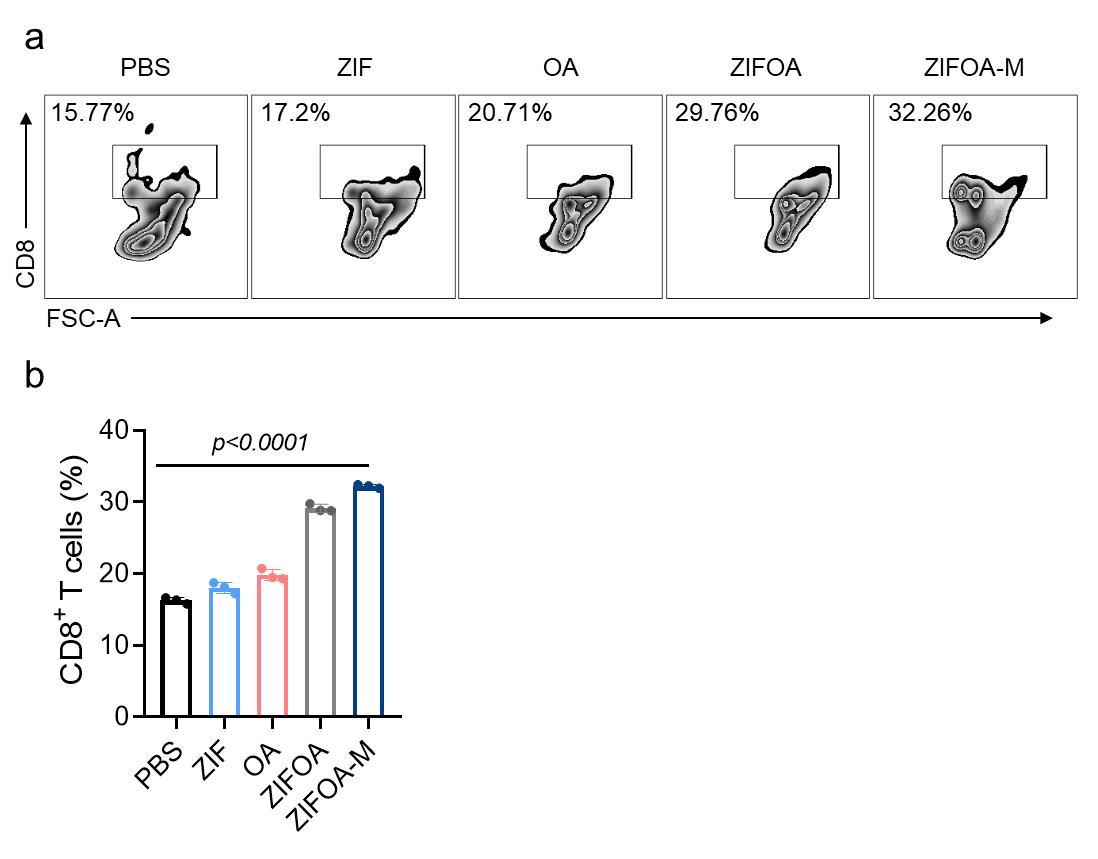


Figure S15. Representative flow cytometry plots (a) of CD8^+^ T cells and statistical results (b) in spleen. Data were expressed as mean ± standard deviations (SD) (n = 3).

Figure S16. Monitoring the survival period of mice treated with the different formulations (n = 5).


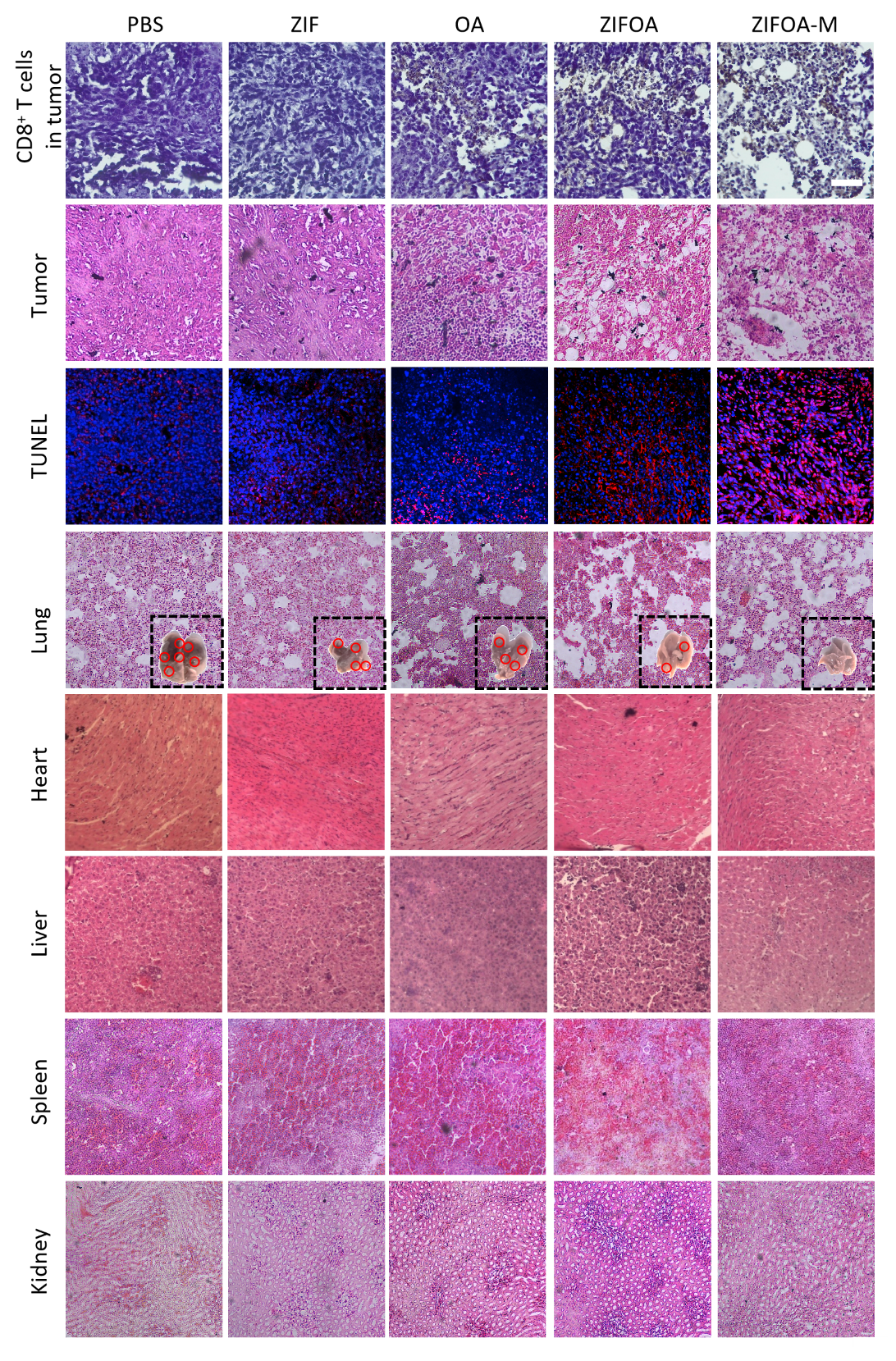


Figure S17. **Immunohistochemical evaluation of different formulations treatment groups.** Immunohistochemical staining of CD8^+^ T cells and apoptotic cells in tumor. H&E staining of tumor, lungs, heart, liver, spleen and kidney. Scale bar: 50 μm.

Figure S18. Quantification of pulmonary tumor nodules in mice following treatment with various therapeutic formulations. Data were expressed as mean ± standard deviations (SD) (n = 3).


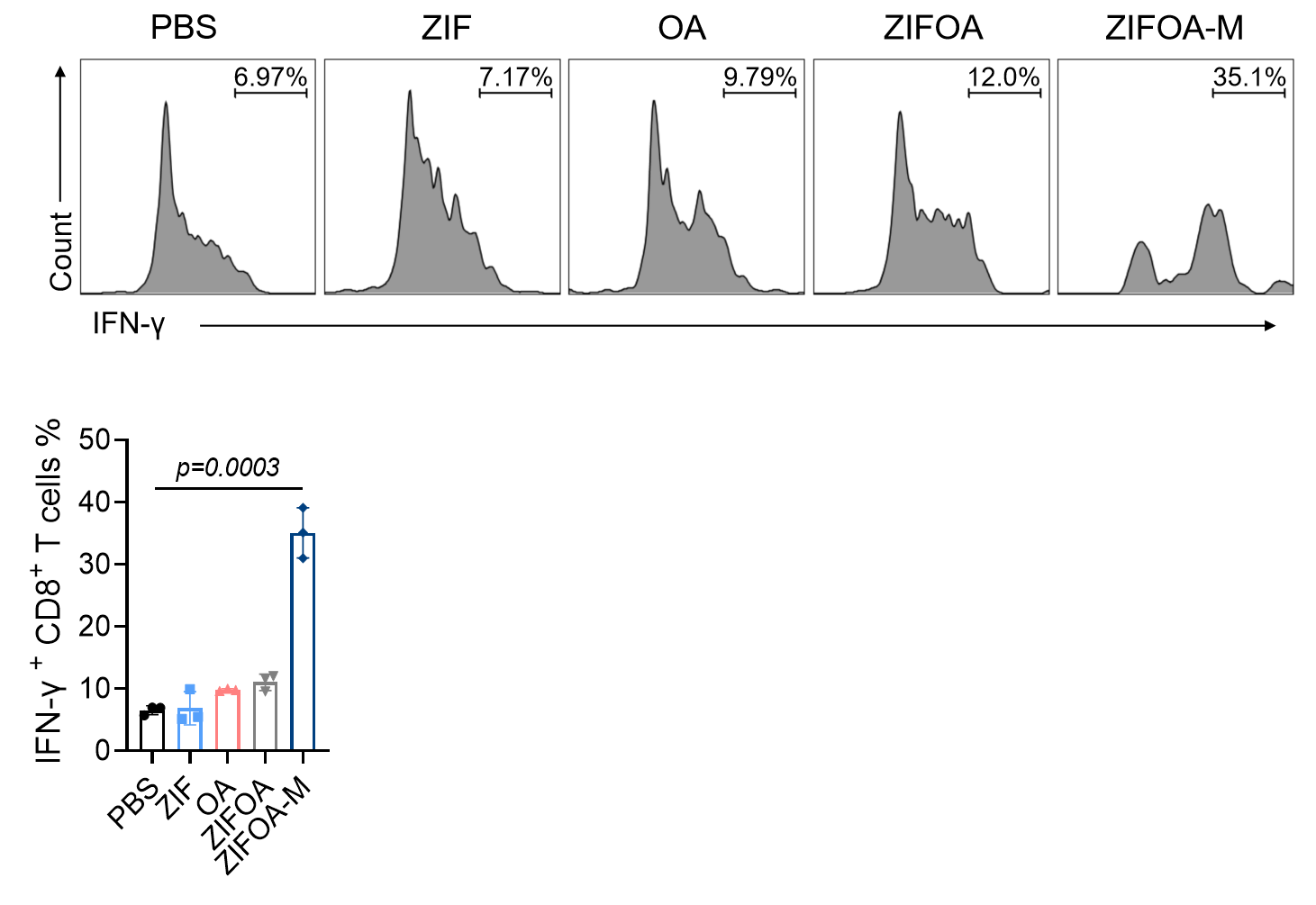


Figure S19. Flow cytometry analysis of IFN-γ^+^ CD8^+^ T cells in tumors following treatment with different formulations. Data were expressed as mean ± standard deviations (SD) (n = 3).
